# Multiple introductions of NRCS-A Staphylococcus capitis to the neonatal intensive care unit drive neonatal bloodstream infections: a case-control and environmental genomic survey

**DOI:** 10.1099/mgen.0.001340

**Published:** 2025-01-07

**Authors:** Emily A. Lees, Jessica Gentry, Hermione Webster, Nicholas Sanderson, David Eyre, Daniel Wilson, Sam Lipworth, Derrick Crook, T.H. Nicholas Wong, Anthony Mark, Katie Jeffery, Stéphane Paulus, Bernadette C. Young

**Affiliations:** 1 Department of Paediatrics, University of Oxford, Oxford, UK; 2 Fitzwilliam College, Cambridge, UK; 3 Department of Microbiology, Oxford University Hospitals, Oxford, UK; 4 Nuffield Department of Medicine, University of Oxford, Oxford, UK; 5 National Institute for Health Research (NIHR) Oxford Biomedical Research Centre (BRC), Oxford, UK; 6 Nuffield Department of Population Health, University of Oxford, Oxford, UK; 7 Department for Continuing Education, University of Oxford, Oxford, UK; 8 Stoke Mandeville Hospital, Buckinghamshire Healthcare NHS Trust, Aylesbury, UK; 9 Department of Neonatology, Oxford University Hospitals, Oxford, UK; 10 Radcliffe Department of Medicine, University of Oxford, Oxford, UK

**Keywords:** antimicrobial resistance, infection control, late-onset sepsis, neonates, *Staphylococcus capitis*

## Abstract

**Background.** The *Staphylococcus capitis* NRCS-A strain has emerged as a global cause of late-onset sepsis associated with outbreaks in neonatal intensive care units (NICUs) whose transmission is incompletely understood.

**Methods.** Demographic and clinical data for 45 neonates with *S. capitis* and 90 with other coagulase-negative staphylococci (CoNS) isolated from sterile sites were reviewed, and clinical significance was determined. *S. capitis* isolated from 27 neonates at 2 hospitals between 2017 and 2022 underwent long-read (ONT) (*n*=27) and short-read (Illumina) sequencing (*n*=18). These sequences were compared with *S. capitis* sequenced from blood culture isolates from other adult and paediatric patients in the same hospitals (*n*=6), *S. capitis* isolated from surface swabs (found in 5/150 samples), rectal swabs (in 2/69 samples) in NICU patients and NICU environmental samples (in 5/114 samples). Reads from all samples were mapped to a hybrid assembly of a local sterile site strain, forming a complete UK NRCS-A reference genome, for outbreak analysis and comparison with 826 other *S. capitis* from the UK and Germany.

**Results.***S. capitis* bacteraemia was associated with increased length of NICU stay at sampling (median day 22 vs day 12 for other CoNS isolated; *P*=0.05). A phylogeny of sequenced *S. capitis* revealed a cluster comprised of 25/27 neonatal sterile site isolates and 3/5 superficial, 2/2 rectal and 1/5 environmental isolates. No isolates from other wards belonged to this cluster. Phylogenetic comparison with published sequences confirmed that the cluster was NRCS-A outbreak strain but found a relatively high genomic diversity (mean pairwise distance of 84.9 SNPs) and an estimated NRCS-A *S. capitis* molecular clock of 5.1 SNPs/genome/year (95% credibility interval 4.3–5.9). The presence of *S. capitis* in superficial cultures did not correlate with neonatal bacteraemia, but both neonates with rectal NRCS-A *S. capitis* carriage identified also experienced *S. capitis* bacteraemia.

**Conclusions.***S. capitis* bacteraemia occurred in patients with longer NICU admission than other CoNS. Genomic analysis confirms clinically significant infections with the NRCS-A *S. capitis* strain, distinct from non-NICU clinical samples. Multiple introductions of *S. capitis*, rather than prolonged environmental persistence, were seen over 5 years of infections.

## Data Summary

Sequence data generated by this study have been deposited in the Short Read Archive (Bioproject PRJEB76591, accession numbers ERS20245094–ERS20245140). All accession numbers are listed in supplementary data. Individual-level data for the case-control study cannot be shared without indirect identification so has not been included.

**Impact Statement**

The NRCS-A strain of *Staphylococcus capitis* has emerged as a globally successful organism in neonatal intensive care units (NICUs). Recent studies have demonstrated that the lineage is widespread throughout the UK and Europe and closely related clusters across the UK suggest inter-regional spread. By integrating clinical and sequencing data, this study demonstrates that most *S. capitis* bacteraemias are true infections and that NRCS-A *S. capitis* infection is specifically associated with more time in NICU compared with other coagulase-negative staphylococcal infections. Genomic analysis reveals that closely related isolates have been detectable in one centre over 6 years. We calculate the ‘molecular clock’ for NRCS-A *S. capitis* from UK and European sequences. This mutation rate, while relatively slow (about 5 SNPs per year) is slightly higher than a rate of 1.7 SNPS per year estimated from an earlier collection of strains 1994–2015. Together, these studies suggest that even closely related isolates (<100 SNPs apart) most likely represent multiple introductions to the NICU, with subsequent onward spread. Control of this infection must therefore focus not only on eradication from the NICU environment but also on continued vigilance and control of spread, as well as reduction in the selection pressures favouring NRCS-A, such as glycopeptide exposure.

## Introduction

An estimated 15 million neonates are born prematurely worldwide, with ~1 million deaths per year relating to complications of prematurity [1]. The increase in survival of extremely premature neonates in higher-income settings has consequently increased the incidence of severe acute and chronic neonatal morbidities [2,4]. Prolonged neonatal intensive care unit (NICU) admissions, along with interventions (such as central lines), increase the risk of late-onset sepsis (LOS; sepsis arising ≥72 h after birth and during NICU admission) [5]. Despite infection prevention and control measures, 20–38 % of babies born prematurely experience at least one episode of LOS, with mortality of up to 19% [6]. Gram-positive organisms cause 70% of neonatal sepsis, dominated by coagulase-negative staphylococci (CoNS) in 47% of infections [5].

Amongst CoNS, *Staphylococcus capitis* is increasingly recognized as a cause of LOS in the NICU [7]. CoNS are a common blood culture contaminant and often not routinely speciated by microbiology laboratories, potentially masking the true prevalence of *S. capitis*. In one French NICU study, LOS due to *S. capitis* in neonates born under 34-week gestation demonstrated an increased composite score of death or serious morbidity compared with LOS due to other CoNS (57 vs 32 %, *P*=0.022) [8]. The first indication that a specific clone of *S. capitis* had spread widely came when 40 *S. capitis* isolates from 7 geographically distant French NICUs were found to cluster genomically. This cluster was initially characterized as the NRCS-A *S. capitis* pulsotype [9]. Further study confirmed clonality [10], and an examination of 154 *S. capitis* strains from 34 NICUs in 17 countries (1994–2015) has found the NRCS-A clone in all of these settings, confirming its concerning global spread [11].

The high prevalence, transmission and sustained presence of a genetically distinct clone in NICUs worldwide suggest the possibility of a specific NICU environmental reservoir. Within NICUs, the NRCS-A *S. capitis* clone has been isolated from almond oil bottles [12], incubators, stethoscopes, mattresses [13,14] and transient carriage on the hands of voluntary caregivers [14]. Despite widespread distribution within the NICU, often within the immediate neonatal bedspace, no single persistent environmental NICU reservoir for NRCS-A *S. capitis* has been identified, and the detection of the NRCS-A clone on the unit is not always associated with clinical cases [15].

Whole-genome sequencing (WGS) of NRCS-A *S. capitis* highlights the acquired mutations in antimicrobial resistance genes, corresponding to antibiotics frequently prescribed in NICU. NRCS-A displays fluoroquinolone susceptibility, beta-lactam and gentamicin resistance and vancomycin resistance or heteroresistance (wherein a subset of an antimicrobial-susceptible bacterial population has decreased susceptibility) [9] and acquires non-reversible vancomycin resistance more rapidly than non-NRCS-A *S. capitis* after vancomycin exposure [16]. NRCS-A differs from other CoNS by displaying *nsr-*encoded resistance to nisin (an anti-staphylococcal bacteriocin expressed by intestinal-colonizing flora), and the international outbreak strains contain a chromosomal cassette carrying heavy metal resistance genes [17]. Specific polymorphisms hypothesized to favour persistence in NICUs include variants in *glnQ* (contributing to vancomycin resistance [18]) and *tarJ* (promoting biofilm formation [19]). Comparison of NRCS-A to other *S. capitis* found in NICU settings has suggested that genes associated with metal transport and acquisition contribute to NRCS-A environmental adaptation [20].

A potential outbreak in UK NICUs was identified by the UK Health Security Agency (UKHSA) in 2021. Eighty *S. capitis* bacteraemia cases were reported in London and the surrounding area with antibiograms similar to NRCS-A clone; these isolates were later confirmed as NRCS-A with WGS, and inter-regional spread of this clone was noted [21,22]. Further large-scale study compared clinical outcomes from CoNS episodes in NICUs in England but did not find differences in survival to discharge or 30-day mortality by CoNS species, including *S. capitis* (NRCS-A and non-NRCS-A lineages were not differentiated in this study) [23].

We sought to understand the clinical significance of *S. capitis* in the culture of blood and other sterile samples from patients in our NICU. We conducted a case-control study on infants with *S. capitis* and other CoNS isolated from sterile sites to determine the likely clinical significance of *S. capitis* isolation. WGS was used to compare *S. capitis* from NICU samples to other *S. capitis* isolates from across two hospitals, to identify whether the NRCS-A clone was present and its likely distribution and to seek evidence of within-unit transmission. We sequenced *S. capitis* isolated from non-sterile sites and an environmental sampling programme to investigate whether at-risk neonates could be identified through screening and identify potential reservoirs in the NICU.

## Methods

### Clinical data

Demographic and clinical data from electronic patient records were reviewed for 45 cases (consecutive neonates on a tertiary NICU in Oxford, UK, with *S. capitis* isolated from blood culture between January 2019 and December 2022) and 90 controls [randomly selected neonates with other CoNS isolated from blood culture over the same time period (Fig. S1, available in the online version of this article)]. Bacterial species were identified by MALDI-TOF MS. Cases and controls were identified from the hospital laboratory records. De-identified data were reviewed by two clinicians. Culture-positive episodes were classified as contaminant, central line-associated bloodstream infection (CLABSI), necrotizing enterocolitis (NEC)-associated bloodstream infection (BSI) or phlebitis with BSI. Infection was defined following Infection in Critical Care Quality Improvement Programme (ICCQIP) [24] and National Healthcare Safety Network criteria [25] (Tables S1–S4). *S. capitis* antibiotic susceptibilities were reported, following automated broth microdilution testing [BD Phoenix™ (BD)], or in one isolate, antibiotic disc diffusion testing.

### *S. capitis* clinical isolates

A total of 31 *S. capitis* isolates collected from samples of usually sterile sites (blood, cerebrospinal fluid and line tips), between 2017 and 2022, were retrospectively identified from a tertiary hospital (Hospital 1), along with 2 from a network hospital (Hospital 2). Samples were from patients cared for in NICU (*n*=27; 24 of whom had matching clinical data reviewed above), an adult intensive care unit (*n*=1) or other adult/paediatric wards (*n*=5).

### Superficial swab screening

Superficial swabs, collected during routine NICU care in Hospital 1 (ear, throat, groin, eye and skin) (*n*=150) between March and June 2022, were cultured onto Columbia blood agar (CBA) and incubated aerobically and anaerobically at 37 °C for 48 h. Any suspected *S. capitis* colonies were identified by MALDI-TOF MS (Bruker) [26].

### Rectal screening programme

Routine rectal screening swabs for surveillance of organisms producing extended-spectrum beta-lactamase from NICU patients received in May 2022 (*n*=69) were inoculated onto MRSA brilliance agar (Thermo Fisher) and incubated aerobically for 5 days as described by Butin *et al*. [27]. Any growth was identified by MALDI-TOF MS.

### Environmental sampling

A total of 114 sites in the NICU were sampled on 1 day in April 2022, using a combination of liquid amies swabs (MWE Ltd) and Petrifilm™ (Thermo Fisher). Locations sampled included sites in and around two clean and two occupied incubators, ward surfaces and high touch points (Figs S2 and S3). Incubator locations were sampled with a swab, Aerobic Culture (AC) Petrifilm™ (3M) and Staph Xpress (SE) Petrifilm™ (3M). Hard-to-reach locations and uneven or fabric surfaces were sampled with swabs. All other locations were sampled with swabs and AC Petrifilm™. Petrifilms and swabs were cultured onto CBA and incubated aerobically at 37 °C for 48 h, and any colonies identified by MALDI-TOF.

### WGS

DNA extracted from pure culture of *S. capitis* isolates (as outlined in Methods S1) underwent sequencing, alongside two reference commensal *S. capitis* strains (DSM67173 and NCTC11045). Short-read sequencing was undertaken using Illumina MiSeq for 18 *S. capitis* isolates from Hospital 1, 2019–2021. All included isolates (*n*=47) underwent long-read sequencing on the GridION platform using R9.4.1 flow cells [FLO-MIN106 (Oxford Nanopore Technologies)].

### Bioinformatic analysis

Complete details of bioinformatic methods can be found in the Supplement. Short-read Illumina sequencing files underwent *de novo* assembly of reads into contigs using Velvet v1.0.18 [28]. An alignment-free phylogeny was constructed using JolyTree [29] to compare assemblies to NRCS-A sequences originating from the UK (CR05 [30]), France (CR01 [31] and CR09 [30]), Belgium (CR03 [30]) and Australia (CR04 [30]), which had been published as shotgun assemblies.

Long- and short-read hybrid assembly was performed using the Dragonflye pipeline v1.1.2 [32], to construct a circularized reference genome for the NRCS-A clade. Nanopore-only consensus sequences were then generated by alignment of reads to the newly created UK NRCS-A *S. capitis* reference genome using minimap2 v2.17-r974-dirty [33], and variants were called using clair v2.2.2 [34]. SNP variant calls were filtered as previously described, removing reads with <10 reads or <80% supporting reads [35,36]. Consensus genomes were used to construct a maximum likelihood (ML) tree constructed from the mapped reads using RaXML v8.2.12 (assuming a general time reversible nt substitution model) [37] and adjusted for recombination using ClonalFrame v1.12 [38].

*S. capitis* sequences (including NRCS-A isolates) in the NCBI Short Read Archive (PRJNA751027 [20] and PRJEB51567 [22], *n*=826) were downloaded and mapped to the UK NRCS-A hybrid reference genome. The cluster of isolates containing local NRCS-A isolates and any strains within 600 SNP distances was identified. In addition to the identification by phylogenetic relationship, isolates in the cluster were screened for the presence of *nsr* gene sequence to identify them as NRCS-A [17,21]. These sequences were reduced to variant-only sites, and an ML phylogeny was generated using IQTREE 2 v2.2.5 [34], following which recombination regions were identified with ClonalFrame and removed [33]. The complete dataset was subsampled to 100 to reduce over-sampling of nodes from outbreak studies, as BEAST depends on an assumption of independent sampling. All local sequences were then re-added. Bayesian coalescent analysis of the NRCS-A-clustered isolates in the resulting recombination-adjusted phylogeny was performed with BEAST v1.10.4 [39] to estimate the mutation rate and produce a time-dated phylogenetic tree (with the most recent sample dated 6 June 2022). Phylogenetic trees were annotated and visualized using the statistical package ‘R’ (v4.3.0) [40] using ggtree v3.8.0 [41].

The impact of sex, gestation, birthweight and age at the sample on the species of bacteria isolated in blood culture (*S. capitis* vs another coagulase-negative staphylococcal species) was modelled using univariable and multivariable logistic regression [including all variables with evidence of impact on the outcome (*P*<0.1)]. We accounted for non-linear effects of continuous variables by using natural cubic splines with up to three default spaced knots, or log2 transformations, choosing the best fitting model, also including a linear model in comparisons, by minimizing the Bayesian information criterion. Case-control study statistical analysis was done using the statistical package R (v4.3.0) [39] using the tidyverse package [41].

## Results

### Majority *of S. capitis* bacteraemia cases are true infections and are associated with the length of time in NICU

Forty-five *S. capitis* cases were compared to 90 CoNS blood culture-positive controls. Amongst controls, *Staphylococcus epidermidis* was the commonest species (42/90; 49%), followed by *Staphylococcus haemolyticus* (33/90; 37%). *Staphylococcus warneri*, *Staphylococcus hominis* and *Staphylococcus saprophyticus* were detected at lower frequencies (Fig. S4a). All identified CoNS species were detected both in episodes adjudicated as likely CLABSI and as likely contaminants (from patients’ commensal flora or other technical sources of contamination) (Fig. S4b).

There was no difference in gender amongst neonatal *S. capitis* cases compared with CoNS controls (53 vs 43% female, univariable odds ratio (OR)=1.17 [95 % confidence interval (CI) 0.78–3.76; *P*=0.15]) (Table 1). *S. capitis* cases occurred in neonates born at earlier gestations (median 26 vs 27 completed weeks (Interquartile range (IQR)24–28 vs 25–33 weeks; OR=0.92 [0.84–0.99; *P*=0.04]). Neonates positive for *S. capitis* had a higher median age at the time of sampling [22 days (IQR 11–38) vs 12 days (IQR 7–23 days), Fig. S5], with the risk of *S. capititis* infection increasing each time length of stay on the unit doubled (OR per doubling of age in days=1.29 [1.01–1.68; *P*=0.05]). There was also marginal evidence that neonates with higher birthweights were at less risk of *S. capitis* (OR per kilogramme increase=0.61 [0.34–0.96; *P*=0.06]). Likely reflecting limited power, no individual factors were independently associated with *S. capitis* in blood culture on multivariate analysis, although point estimates for each effect remained similar, albeit with some attenuation in the association with birthweight.

**Table 1.** Comparison of blood culture-positive *S. capitis* cases and CoNS controls

	*S. capitis*, *n*=**45**	CoNS, *n*=**90**	Univariate analysis[OR (95% CI)]	***P*-value**	Multivariateanalysis[OR (95% CI)]	***P*-value**
Female, *n* (%)	24 (53%)	36 (43%)	1.71 (0.78–3.76)	0.15^*^	na	na
Birthweight (OR per kg increase), median [IQR]	0.80 [0.65–1.2]	0.84 [0.7–1.5]	0.61 (0.34–0.96)	0.06^^^	0.85 (0.33–2.05)	0.7^
Gestation (OR per completed week), median [IQR]	26 [24–28]	27 [25–33]	0.92 (0.84–0.99)	0.04^^^	0.96 (0.82–1.12)	0.6^
Age at positive culture (OR per doubling of age in days), median [IQR]	22 [11–38]	12 [7–23]	1.29 (1.01–1.68)	0.05^^^^	1.26 (0.84–1.94)	0.3^^

Key – sStatistical analysis method: *Fisher’s test, ^ linear model, ^^log2 -transformed model, na not tested.

Mortality at 14 days did not differ between *S. capitis* cases and CoNS controls [3/45 (6.7 %) vs 4/90 (4.4 %), *P*=0.69, Fisher’s exact test]. In each group, there was one infant whose death was possibly related to staphylococcal BSI [1/24 (4.2 %) vs 1/34 (2.9 %), *P=*1, Fisher’s exact test].

Amongst all infants with positive blood cultures, there was marginal evidence that *S. capitis* bacteraemia cases were more likely to be classified as true infection [53 vs 38 % (*P*=0.09)] rather than detected as a contaminant (Table 2). Infection cases demonstrated similar proportions of attributable source compared with CoNS controls.

**Table 2.** Classification of blood culture results according to the likely presence of infection and source

Classification of blood culture	*S. capitis*, *n* (%)	CoNS, *n* (%)	***P*-value**
Contaminant	21 (47)	56 (62)	0.09*
Infection	24 (53)	34 (38)	
Infection according to source			
CLABSI	18 (40)	29 (32)	0.6**
NEC/translocation-associated BSI	5 (11)	4 (4)	
Phlebitis+BSI	1 (2)	1 (1)	

Key - sStatistical analysis method: *Cchi-squared, **Cchi-squared with 2 degrees of freedom.

When only cases and controls classified as infections were compared, there was marginal evidence of lower birthweight and earlier gestation in *S. capitis* cases (*P*=0.1), which was not seen in multivariable analysis (Table 3). An increasing number of days since birth were seen in those with the finding of *S. capitis* infection compared to other CoNS on both univariable and multivariable logistic regressions. Days from birth at blood culture result were independently associated with *S. capitis* infection, with a doubling of age showing increased OR 1.90 (95% CI 1.10–3.50, *P*=0.02).

**Table 3.** Comparison of clinically adjudicated infection with *S. capitis* (cases) and CoNS (controls)

	*S. capitis*, *n*=**24**	CoNS, *n*=**34**	Univariate analysis [OR (95% CI)]	***P*-value**	Multivariate analysis [OR (95% CI)]	***P*-value**
Female, *n* (%)	12 (50%)	14 (41%)	1.4 (0.44–4.65)	0.6*	na	na
Birthweight (OR per kg increase), median [IQR]	0.8 [0.65–1.23]	0.88 [0.78–1.27]	0.36 (0.09–1.1)	0.1^	0.77 (0.06–9.62)	0.8^
Gestation (OR per completed week), median [IQR]	26 [24–27]	26 [25–32]	0.88 (075–1.02)	0.1^	0.90 (0.65–1.26)	0.6^
Age at positive culture (OR per doubling of age in days), median [IQR]	21 [11–33]	11 [8–15]	1.93 (1.13–3.53)	0.02^	1.90 (1.10–3.50)	0.02^^

Key – sStatistical analysis method: *Fisher’s test, ^ linear model, ^^log2 -transformed model, na not tested.

### *S. capitis* antibiotic susceptibilities consistent with NRCS-A clone

**Fig. 1.**
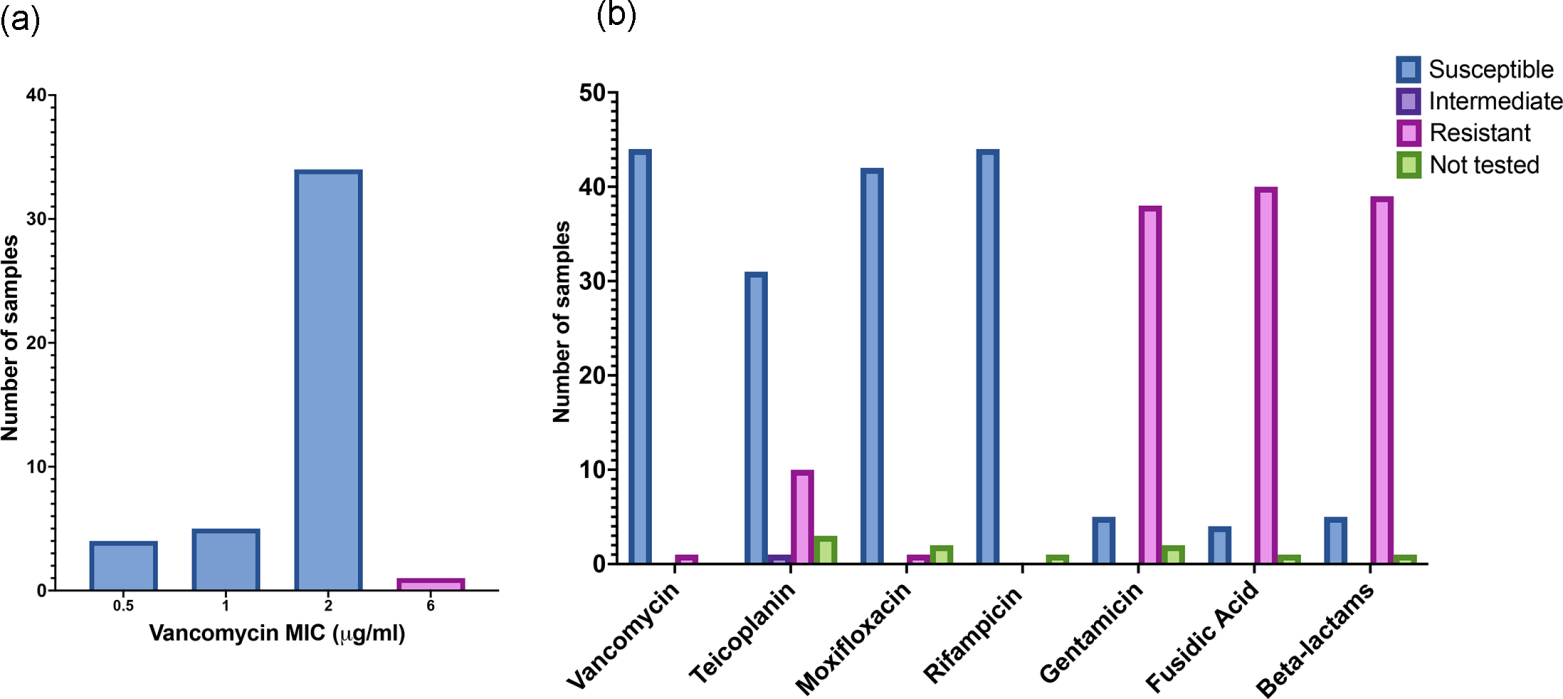
(**a**) Vancomycin MIC and (**b**) antibiograms with available results from *S. capitis* clinical isolates, demonstrating a pattern consistent with that seen for the NRCS-A clone (fluoroquinolone and rifampicin susceptibility, beta-lactam, fusidic acid and gentamicin resistance and vancomycin heteroresistance).

Amongst clinical *S. capitis* isolates, 44/45 (98%) were reported vancomycin susceptible and 1/45 (2%) resistant [minimum inhibitory concentration (MIC) 6 µg/ml] (Fig. 1a). MIC was determined for 44/45 cases, and these clustered close to the breakpoint (≤4 µg/ml) [42]: 4/44 (9%) had MIC=0.5 µg/ml, 5/44 (11%) had MIC=1 µg/ml and 34/44 (77%) had MIC=2 µg/ml.

Teicoplanin susceptibility was reported for 42 cases with 31/42 (74%) susceptible, 1/42 (2%) intermediate and 10/42 (24%) resistant. Isolates were predominantly resistant to fusidic acid (40/44; 91%), gentamicin (38/43; 88%) and anti-staphylococcal beta-lactams (inferred from flucloxacillin susceptibility) (39/44; 89%). Isolates were almost universally susceptible to rifampicin (44/44; 100%) and moxifloxacin (42/43; 98%) (Fig. 1b).

**Fig. 2.**
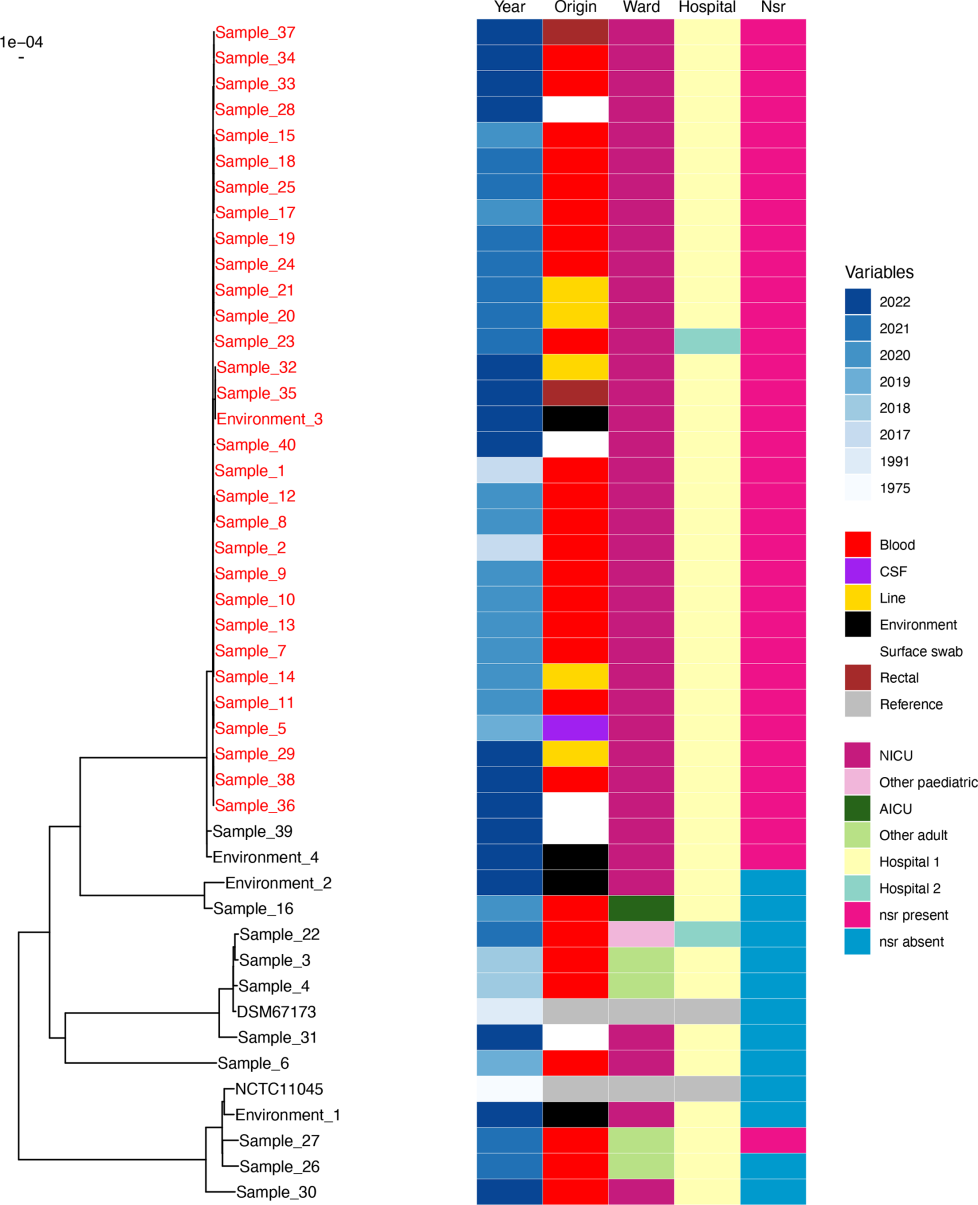
ML phylogeny of *S. capitis* isolates from two hospitals including isolates from non-NICU settings in both hospitals. Year of isolation, clinical origin, ward location hospital and presence of the NRCS-A marker *nsr* gene are denoted in legend. Samples in the local NICU cluster are labelled in red, and non-cluster samples are labelled in black. CSF, cerebrospinal fluid; Line, vascular line tip; AICU, adult intensive care unit. Scale bar represents an evolutionary distance of 10^−4^ (one ten thousandth of the genome length, ~260 bp).

The four samples with the lowest vancomycin MIC (0.5 µg/ml) were also susceptible to gentamicin and beta-lactams, and 2/4 were susceptible to fusidic acid, suggesting a small number of phenotypically different *S. capitis* not typical of the NRCS-A clone [7]. All of these four samples were clinically adjudicated as contaminants. Two were collected on the first day of life: subsequent sequencing confirmed these two samples to be distinct from the NICU NRCS-A cluster (Fig. 2, samples 6 and 30). It is therefore likely that the findings related to clinical infection (Table 3) represent demographics specifically associated with NRCS-A *S. capitis*.

### *S. capitis* NRCS-A clone found in the NICU demonstrates significant variation

In order to assess whether the NRCS-A clone was present in our setting, genomes of 18 NICU *S. capitis* isolates from sterile site cultures in Hospital 1 were compared with publicly available shotgun assemblies of NRCS-A *S. capitis* from the UK (CR05), France (CR01 and CR09), Belgium (CR03) and Australia (CR04), in addition to 2 *S. capitis* reference strains: DSM67173 and NCTC11045 [43] (Table S5). Hospital 1 NICU genomes formed a cluster of limited diversity (Fig. S6). The most recent common ancestor of the NICU isolates sequenced in this study was most closely related to the UK NRCS-A strain, followed by NRCS-A strains from France, Belgium and Australia, respectively (Fig. S6). Hybrid assemblies representative of the UK NRCS-A strain were constructed using short- and long-read sequencing of three isolates obtained in this study, with a fully circularized chromosome recovered in all three (length 2 577 781–2 612 053 bp) and up to three smaller circularized contigs consistent with variably present plasmids 18–59 kb in length. The longest contigs (representing bacterial chromosome) showed preserved homology with no evidence of structural rearrangement when examined with Mauve (v2.4.0) [44]. The earliest of these (sample 15, 2020) was used as a UK NRCS-A reference for further analysis.

Consensus whole genomes for 47 *S. capitis* isolates from multiple locations across 2 hospitals sequenced by ONT were constructed by mapping to the completed UK NRCS-A reference strain. Median coverage with least 10 reads depth was 99.7% (IQR 91.8–99.9%). One sample (isolated from a stethoscope in the NICU environment) showed sequence coverage of <50% and was excluded from further analysis. Consensus genomes were used to construct an ML phylogeny of local *S. capitis* adjusting for recombination. This demonstrated that 25/27 NICU *S. capitis* sterile site isolates formed a cluster, along with 3/5 superficial, 2/2 rectal and 1/5 environmental NICU isolates (Fig. 2). The mean pairwise distance estimated from ML tree branch length within this cluster corresponded to 84.9 whole-genome SNPs (wgSNPs), compared with pairwise distance across the sample set of 30 094 wgSNPs. The only NICU sterile site genomes not within this cluster were from blood cultures collected on the first day of life (samples 6 and 30). Clinical review had classified these as contaminants, most likely representing colonization with maternal flora, rather than healthcare-associated infection. *S. capitis* genomes from NICUs at both hospitals formed part of the NICU cluster. No samples from adults or older children belonged to the NICU cluster. Environmental and superficial samples from NICU patients were found both within and outside the NICU cluster. The majority (75%) of environmental *S. capitis* isolates identified in NICU were unrelated to the cluster. All isolates in the cluster contained the NRCS-A-specific marker gene *nsr* [17].

**Fig. 3.**
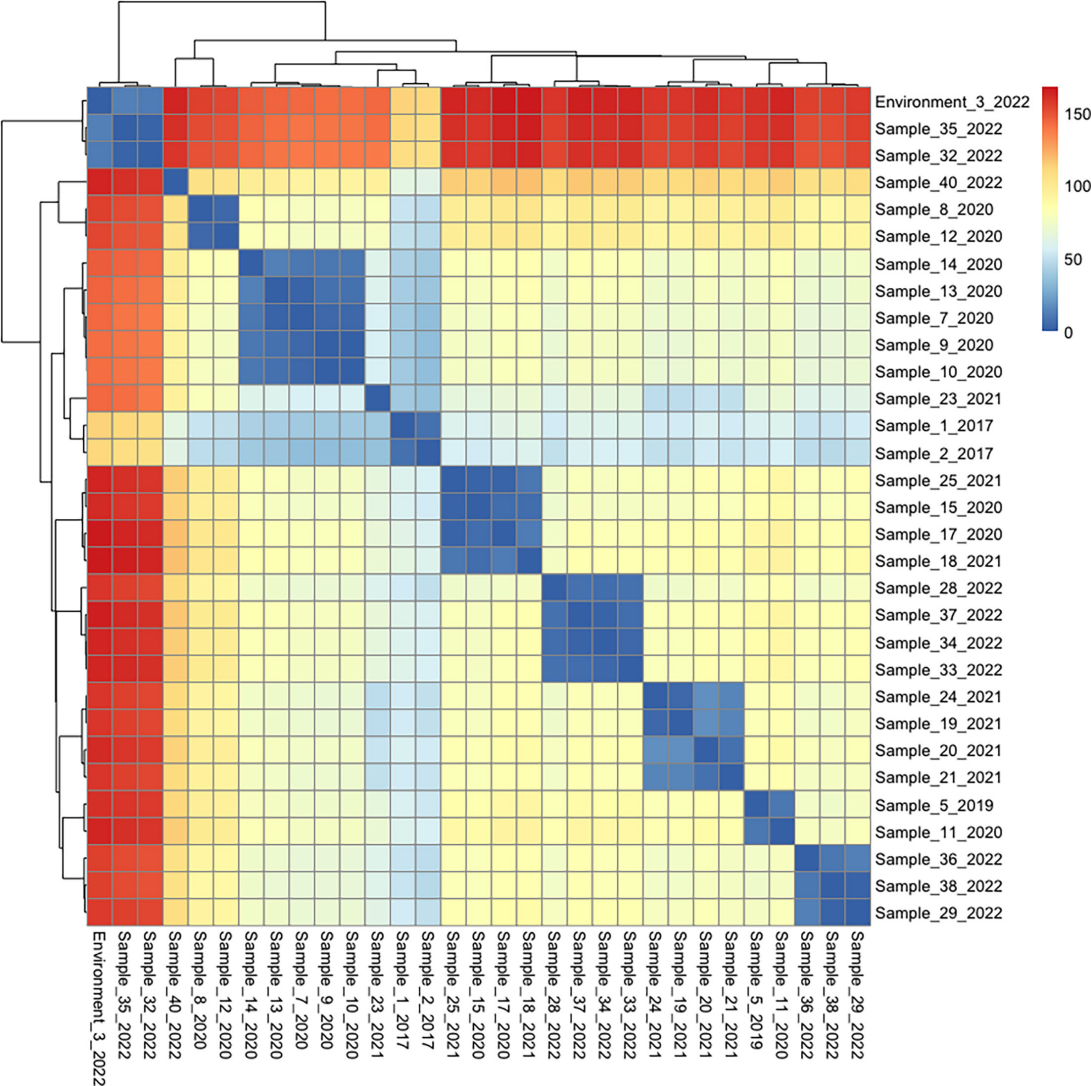
Pairwise distance matrix of 31 closely related *S. capitis* strains from clinical and environmental samples found in NICU (all confirmed as NRCS-A clone). Samples 32 and 35 are from the same patient (line tip culture and rectal swab culture). Samples 34 and 37 are from the same patient (blood culture and rectal swab culture).

Having confirmed the NICU cluster as NRCS-A (Fig. S6), we examined the phylogenetic relationship of clustered isolates more closely for evidence of local transmission. Pairwise distances were estimated by branch lengths on ML phylogeny after correction for recombination (Fig. 3). ONT reads mapped to the UK NRCS-A reference genome gave good resolution of the NRCS-A isolates, with a median 99.8% of the genome called (after variant filtering) for isolates in the cluster. The observable diversity across the whole genome showed that sub-groups of up to 5 isolates showed very close relationships [with pairwise distances of 2–12 SNPs across the whole genome (wgSNPs)]. Relatively longer distances (70–167 wgSNPs) separated the majority of 2022 genomes from earlier strains and from each other. By 2022, four distinct sub-clades are observed. The Hospital 2 NICU genome (sample 23, 2021) had pairwise distances of 35.9 and 40.1 wgSNPs with its nearest neighbours (samples 1 and 2), isolated in 2017. This patient had not previously been admitted to Hospital 1. The close genomic relatedness would be consistent with previous (unobserved) between-unit transmission of the clone.

A time-dated phylogeny of 717 local and publicly available *S. capitis* sequences was constructed, including NRCS-A and any clustering within 1000 SNPs before recombination correction. This reveals five distinct clusters in local NICU isolates, whose most recent common ancestors are most likely dated to July 2007 [95% credibility interval (CrI) July 2006–July 2008) (Fig. S7). This tree was trimmed to 100 sequences, to avoid oversampling from outbreaks, and used to estimate the NRCS-A evolutionary rate using BEAST (Fig. S8). This phylogeny displayed a linear relationship between the phylogenetic distance and the SNP distance between each sample pair (Fig. S9) in the recombination-adjusted phylogeny. A molecular clock rate derived from this phylogeny gave a rate calculated at 2.00×10^−6^ or 5.1 SNPs per genome per year (95% CrI 4.3–5.9 SNPs/genome/year).

These observations support the hypothesis that multiple NRCS-A lineages were introduced to local NICUs over the study period.

### Environment, skin and gastrointestinal carriage may all contribute to NRCS-A *S. capitis* spread and persistence within the NICU

Environmental sampling demonstrated 57 of 114 sites with bacterial growth, with *S. capitis* from 5 locations (Fig. S3): doctors’ pagers, a clean incubator door handle, a patient notes trolley, the handle of a cupboard and a stethoscope held within an in-use incubator. Long-read sequencing of the five *S. capitis* isolates demonstrated that one (clean incubator door handle) was NRCS-A (Fig. 3) and was close to two clinical isolates (samples 32 and 35) from the same patient, being ~11.2 and 13.0 SNPs apart in mean pairwise distance. These samples were collected 15 and 25 days after the environmental isolate. It was not possible to establish an epidemiological link between the incubator and infected neonate. *S. capitis* was also isolated from a trolley containing medical notes; a location not in direct contact with patients, however, is a touch point for NICU staff. After adjustment for recombination, the notes trolley genome had a mean pairwise distance of 532.2 wgSNPs from genomes to the cluster, compared with the mean pairwise distance of 84.9 wgSNPs for genomes within the most closely related NICU isolates (Fig. S10).

Five *S. capitis* isolates were obtained from routinely collected NICU superficial skin swabs (*n=*150; including skin, nose, ear, groin, eye and throat). *S. capitis* was only isolated from ear swabs. WGS demonstrated that 3/5 isolates fell within the NRCS-A cluster (Fig. 2); however, no neonates with superficial colonization had an observed *S. capitis* bacteraemia at any time.

Sixty-nine surveillance rectal swabs from 47 NICU patients were cultured on MRSA brilliance agar, of which 35/69 (51%) demonstrated bacterial growth. Organisms included *S. haemolyticus*, *Bacillus* spp., *Enterococcus faecalis*, *S. epidermidis* and *S. capitis*. Two *S. capitis* isolates were identified from rectal screening cultures, and WGS confirmed that these were NRCS-A outbreak strain (Fig. 2). Both neonates with NRCS-A *S. capitis* identified on rectal culture developed an NRCS-A *S. capitis* BSI either pre- or post-rectal culture. Patient 1 had *S. capitis* BSI 14 days post-admission, with the positive rectal culture identified 14 days after bacteraemia. However, the neonate also had negative rectal cultures at days 6, 17 and 24 following *S. capitis* bacteraemia. Patient 2 had *S. capitis* BSI 31 days after admission, with *S. capitis* identified on rectal culture on day 8 of admission. This neonate also had negative rectal culture 15 days post-bacteraemia episode (Fig. S11).

## Discussion

We report the persisting identification of NRCS-A *S. capitis* in Hospital 1’s NICU over a 6-year period from 2017 to 2022 and possible inter-unit transmission of this pathogen; a closely related strain was recovered from a blood culture sample from a neonate in Hospital 2 who was never admitted to Hospital 1. Our clinical case-control study reveals an independent association between length of time in NICU and *S. capitis* infection, and most episodes of *S. capitis* bacteriemia are likely true infection. Environmental sampling alongside rectal and superficial site cultures demonstrates the presence of the NRCS-A clone within the NICU environment, as well as colonizing neonatal skin and gut. No clinical cases of infection with the NRCS-A clone were observed outside the NICU, indicating that the identification of NRCS-A *S. capitis* in the NICU is not solely due to ascertainment bias as a result of the UKHSA request to speciate and report this organism in neonatal samples [21].

Genetic diversity is observed within the NRCS-A clone even in this single location. While close neighbours (<50 wgSNPs) within the cluster were found in clinical or environmental samples for most isolates, the clusters were limited to 5 or fewer cases (Fig. 3). Our estimated rate for NRCS-A *S. capitis* is 5.1 SNPS/genome/year, which is consistent with rates for *S. aureus* of 8–10 SNPs/year and *S. epidermidis* mutation rates [45,46]. Allowing for within-host diversity, thresholds of 25–40 wgSNPs have been determined based on WGS to identify recent transmission in *S. aureus* [47,48]. A recent observational study of likely *S. capitis* clusters found that these were related by <50 core genome SNPs [22]. Previous study based on global strains of NRCS-A *S. capitis* between 1994 and 2015 estimated a mutation rate of 1.7 SNPs/genome/year [17]. Evolutionary analysis presented here including strains up to 2022 also finds that the NRCS-A clone accumulates mutations relatively slowly, but we estimate a rate somewhat higher than the previous study at 5.1 SNPs/year. These findings support multiple introductory events with secondary transmission being responsible for the prevalence of *S. capitis* NRCS-A in the NICU and suggest that quite stringent SNP thresholds are required to support recent transmission. The degree of variation observed is not consistent with intra-unit mutations acquired over a 6-year period and sustained transmission from an ongoing unidentified point source.

Extremely premature infants are likely to have prolonged admission to the NICU and therefore have an increased risk of LOS and NEC, as well as higher requirement for prolonged vascular access. In our case-control study, gestational age and weight showed marginal evidence of increasing odds of *S. capitis* infection, while increased age at sampling was specifically associated with *S. capitis* isolation rather than another CoNS species. This was true both in samples classed as contaminants (consistent with colonization) and representing infection. This suggests that the acquisition of *S. capitis* relates to time spent in the NICU. No single environmental site has been implicated in the persistence of NRCS-A *S. capitis* in NICUs [7,14]. In a single day environmental study, we were able to isolate *S. capitis* from high touch points in the unit, but the local outbreak strain was only recovered from a clean incubator. This may reflect failure to remove by cleaning, or re-contamination following cleaning, which was performed according to the manufacturer’s instructions [49]. This isolate was also closely related to sterile site clinical samples taken 15 days later, consistent with either transmission from the environment to the patient or vice versa.

Compared with other CoNS, we found that a larger proportion of *S. capitis* was isolated in the context of an NEC/translocation-associated BSI. This difference did not reach statistical significance in a small study but is notable given the finding of *S. capitis* rectal carriage. No infants with NRCS-A *S. capitis* in superficial skin culture developed bacteraemia, while both infants with NRCS-A *S. capitis* rectal carriage also had bacteraemia. Rectal colonization may be the cause or consequence of BSI. Previous studies have proposed the gut as a reservoir for *S. capitis* infection, with a Taiwanese study on *S. capitis* bacteraemia finding associations with the need for total parenteral nutrition despite no proven cases of CLABSI [50]. Others have also reported * S. capitis* BSI associated with the development of NEC, suggesting gut translocation of the organism [7,51]. A prospective study of 229 neonates found that 36% infants were positive for *S. capitis* in stool culture at some point during their NICU stay; however, *S. capitis* gut colonization was not an independent risk factor for *S. capitis* LOS [52], and a UK/German study demonstrated rectal NRCS-A colonization without associated LOS [20]. While the gut may be one reservoir for *S. capitis*, colonization alone does not induce LOS, and another factor is required.

One such likely factor is antibiotic selection pressure. In addition to high vancomycin MIC, NRCS-A strains demonstrate resistance to aminoglycosides, commonly used antimicrobials for early onset sepsis and LOS in UK NICUs [53]. Resistance – or heteroresistance – to glycopeptides also increases the risk of treatment failure with first-line antimicrobials. Further, *S. capitis* demonstrates adaptation under vancomycin selective pressure when exposed to subtherapeutic concentrations [16]. These observations highlight the urgent need for antimicrobial stewardship in the NICU and the need for neonatal pharmacokinetic data to inform the use of alternative agents appropriate for *S. capitis* (such as linezolid or daptomycin). Additionally, clinicians should improve existing pharmacokinetic evidence on vancomycin in neonates, such as the use of continuous infusions, given the frequent difficulties in achieving therapeutic levels and informing optimal target levels in this population [54].

Our study is limited by the extent of sampling: not all clinical isolates were available for sequencing, and the environmental survey was a point survey rather than a longitudinal study. There is uncertainty in assigning the significance of positive microbiology in neonates, when clinical signs may be non-specific and difficult to interpret. We used ICCQIP definitions, which are less likely to underestimate CLABSI incidence in this population [24]. Antibiotics are often commenced immediately after sampling in premature neonates, due to a high index of suspicion of infection. Thus, repeat positive blood culture (required in CDC case definitions for a skin commensal to be classified as an infectious agent) is often impossible to ascertain, and ICCQIP guidelines allow for this.

This study nevertheless demonstrates the practical impact of WGS for infection prevention and control. The identification of clinically important infections following likely within-unit transmission of NRCS-A *S. capitis* has led to a review of infection prevention practices in our setting, including the method of incubator decontamination (as per UKHSA guidance [55]), enhanced environmental cleaning, education on hand hygiene, correct use of personal protective equipment and a CLABSI prevention care bundle. Chlorhexidine baths are a potential intervention, reducing CLABSI rates in a Canadian NICU study [56]; however, these interventions are not possible in low birthweight infants less than 28 weeks [57] and may not be effective given the potential chlorhexidine tolerance of the *S. capitis* NRCS-A clone (possibly mediated by an SNP in the *qac* gene [13] favouring environmental persistence [58]).

This investigation into *S. capitis* BSIs in an NICU setting demonstrates that *S. capitis* isolates from clinical NICU samples occurred after a longer duration of stay in the NICU. We identified the NRCS-A clone in clinical samples, superficial skin and rectal cultures as well as in the environment over 5 years across two network hospitals. Genomic examination of the outbreak is most consistent with repeated introductions to the environment over this time, rather than the sustained presence of environmental sources, informing the targets of infection prevention interventions. The persistence of the outbreak globally has important implications for judicious antimicrobial stewardship and highlights the need for accurate pharmacokinetic data for antibiotics used in the NICU. Further study is needed to establish the role of asymptomatic carriage in the outbreak, to better inform infection prevention practices, such as screening, and support effective antimicrobial-prescribing decisions in those neonates at risk of infection.

## supplementary material

10.1099/mgen.0.001340 Uncited Supplementary Material 1.

## Abbreviations

AC: Aerobic Culture
BSI: bloodstream infection
CBA: Columbia blood agar
CI: confidence interval
CLABSI: central line-associated bloodstream infection
CoNS: coagulase-negative staphylococci
CrI: credibility interval
ICCQIP: Infection in Critical Care Quality Improvement Programme
IQR: Interquartile range
LOS: late-onset sepsis
MIC: minimum inhibitory concentration
ML: maximum likelihood
NEC: necrotizing enterocolitis
NICU: neonatal intensive care unit
OR: odds ratio
UKHSA: UK Health Security Agency
WGS: whole-genome sequencing
wgSNPs: whole-genome SNPs
