# Supplementary material for: Multiple introductions of NRCS-A Staphylococcus capitis to the neonatal intensive care unit drive neonatal bloodstream infections: a case-control and environmental genomic survey
Source: Microb Genom. 2025 Jan 7;11(1):001340. doi: 10.1099/mgen.0.001340 (PMC11706212; doi:10.1099/mgen.0.001340)
Supplement: Uncited Supplementary Material 1. [file mgen-11-01340-s001.pdf]

Supplementary materials

Methods **pp i-ii**
Tables **pp iii- vi**
- Supplementary tables S1-S5

Figures **pp vii - xviii**
- Supplementary figures S1-S11

References **pp xix-xxi**

Supplementary Methods

Whole genome sequencing

All isolates were sub-cultured on CBA and fresh growth after 24 hours incubation was utilised from DNA extraction using the QuickGene DNA extraction kit (Autogen) as per the manufacturer's instructions with the addition of a mechanical lysis step at 6 m s^{-1} for 40 s (FastPrep, MP Biomedicals). Long fragment extraction was performed on a small selection (n = 4) of isolates to yield the longest possible fragments for ONT sequencing. Long fragment extraction carried out using QIAGEN Genomic tips 100/G (QIAGEN) following the manufacturer's instructions.[1]

Short-read sequencing was undertaken using Illumina MiSeq for 18 *S. capitis* isolates from Hospital 1, 2019-2021. DNA libraries were prepared as per the manufacturer's instructions.[2] Performed using unique dual indexes, 10µl of pre-paired i7 and i5 index adapters were added to each sample. Proceeding pooling of the libraries, DNA quantification was carried out using a Qubit™ 4 Fluorometer (ThermoFisher Scientific, MA, USA) utilising Qubit™ dsDNA BR Assay Kit (Invitrogen, California, USA) and library quality was checked using Agilent 4200 TapeStation system (Agilent Technologies Inc., California, USA) for gDNA. The molarity of the DNA library was calculated and used to dilute the library to a starting concentration of 4nM. The DNA library was diluted and denatured following Illumina guidelines[3] utilising Protocol B for "Bead Based Normalization". A PhiX control was added to the denatured diluted library; the PhiX control was denatured to a final concentration of 20pM before addition of 30µl of PhiX to 570µl of library to achieve >5% spike in, optimal for low diversity libraries.[2]

All included isolates were sequenced on the GridION platform for long read sequencing. DNA concentration was quantified using the Qubit™ 4 Fluorometer (Thermo Fisher) using Qubit™ dsDNA BR Assay Kit (Invitrogen), following the manufacturer's protocol. DNA extracts were multiplexed for 8-10 samples per flowcell using the Oxford Nanopore Technologies (ONT) Rapid Barcoding kit (SQK-RBK004) according to the manufacturer's protocol. Sequencing was performed for 72 hours using the ONT GridION platform (R9.4.1 flow cells; FLO-MIN106). Local base calling completed with Guppy v5 and demultiplexed in real-time.[4]

Bioinformatic analysis

Short-read Illumina sequencing files were processed through an in-house pipeline; COMPASS[5] and *de novo* assembly of reads into contigs using Velvet v1.0.18[6], using default settings and with Velvet Optimiser v.2.1.7 to select kmer lengths for individual sequences.

Assemblies from short read sequencing were compared to published assemblies of NRCS-A sequences originating from the UK (CR05[7]), France (CR01,[8] CR09[7]), Belgium (CR03[7]) and Australia (CR04)[7] by constructing an alignment-free phylogeny using JolyTree.[9]

Long and short read hybrid assembly was performed using the Dragonflye pipeline,[10] to construct a circularised reference genome for the NRCS-A clade. This assembly pipeline used Flye[11] to assemble long reads, and Racon[12] to polish. The assembly was further polished using short reads via Polypolish[13] then Pilon.[14]

Raw ONT fastq files were generated with Guppy v5 (ONT, UK[4]) and pre-processed to remove any human reads with CRuMPIT[15] using centrifuge v1.0.4.[16] Consensus sequences were generated by alignment of reads to the newly created NRCS-A *S. capitis* reference genome using minimap2 v2.17-r974-dirty[17] and variants were called using clair v2.2.2.[18] Variant calls were filtered using a trained random forest classifier. In addition, variants with fewer than 10 reads support or <80% support were masked as previously described.[19,20] Consensus genomes were used to construct a maximum likelihood tree constructed from the mapped reads using RaXML (assuming a general time reversible (GTR) nucleotide substitution model),[21] and adjusted for recombination using Clonal Frame.[22]

Raw Illumina fastq files for 826 NRCS-A clones were downloaded from the NCBI short read archive (PRJEB51567[23] and PRJNA751027[24]) and mapped to the hybrid reference genome with the SNIPPY pipeline.[25] Positions with fewer than 10 reads and less than 80% support for the majority base were masked with N, a full nextflow workflow has been shared.[26] The dataset was then subsampled to 100 samples using treemmer (v0.3).[27] to reduces over-sampled nodes, before all local samples were added back in. Both the full and reduced sequence sets were clustered to a threshold of 600 SNPs difference. For each cluster the alignment and reduced recombination corrected alignment was generated with runListCompare (v0.3.8).[28] This produces an alignment of only variant sites with IqTree v2.2.6,[29] then regions of recombination are removed after detection with clonalframeML.[22] Evolutionary analysis of this alignment was performed with BEAST v1.10.4[30] using a strict clock rate, HKY substitution model, and 5 million MCMC iterations. The analysis was repeated with four different seeds and logs were combined with logcombiner v2.7.6 from the BEAST2 package[31] and manually visualised with Tracer v1.7.2 (within the BEAST package) to inspect convergence. A nextflow workflow for this process is shared.[32]

Statistical analysis:
Continuous variables were compared using Mann-Whitney test with a two-tailed p-value of <0.05 considered statistically significant. Categorical variables are presented as percentages and proportions were compared using Chi-square tests, with a two-tailed p-value of <0.05 considered statistically significant. Statistical analysis was done using GraphPad Prism version 9.5.0.[32]

**a)** A recognised pathogen from at least one culture<br><br>**OR**<br><br>**b)** A common skin microorganism* is cultured from blood<br>AND<br>Patient has ONE of:<br>C-reactive protein >20 g/dL<br>Immature/total neutrophil ratio (I/T ratio) >0.2<br>Leukocytes <5 x 10*9/L<br>Platelets <100 x 10*9/L
**AND**
At least TWO of:<br>Temperature >38°C or <36.5°C or temperature instability<br>Tachycardia or bradycardia<br>Apnoea<br>Extended capillary refill time<br>Metabolic acidosis<br>Hyperglycaemia<br>Other sign of BSI such as apathy

Table S1: Case definition for bloodstream infections in neonates (<28 days of age):
Definitions adapted from ICCQIP definitions of bloodstream infection and CLABSI in neonates[34]

**Aerococcus Sp., Bacillus sp. other, Corynebacterium sp., Coagulase-negative staphylococci not specified, Coagulase-negative staphylococci other, Micrococcus sp., Propionibacterium sp., Staphylococcus Epidermidis, Staphylococcus Haemolyticus, Streptococcus (Viridans group)*

**Meets ALL of the following criteria:**
a) Patient has recognised pathogen or common skin commensals* from 1 or 2 positive cultures
**AND**
• No signs and symptoms (or insufficient to meet case definitions)<br>• No treatment for the positive blood culture[§]

Table S2: Case definition for contaminant in blood culture: Definitions adapted from ICCQIP definitions of bloodstream infection and CLABSI in neonates[34]

[§]In neonatal practice, antibiotics are frequently started presumptively given the risk of deterioration in these patients, therefore this was defined as the decision to discontinue antibiotics by the clinical/Infectious Diseases team without completion of a full course of treatment.

Meets ALL of the following criteria:	
**a)**	One of the criteria for bloodstream infection
**AND**	
**b)**	The presence of at least one central venous catheter at the time of the positive blood culture, or CVC removed within 48 hours before the positive blood cultures
**AND**	
**c)**	The signs and symptoms, and the positive laboratory results, including pathogen cultured from the blood are not primarily related to an infection at another site

Table S3 Case definition for central line-associated bloodstream infection (CLABSI):
Definitions adapted from ICCQIP definitions of bloodstream infection and CLABSI in neonates[34]

Meets ALL of the following criteria:	
**a)**	One of the criteria for bloodstream infection
**AND**	
**b)**	The patient meets criteria for diagnosis of NEC 48 hours prior to or following BSI

Table S4 Case definition for defining necrotising enterocolitis (NEC)/translocation-associated BSI:
Definition adapted from National Healthcare Safety Network guidance on identifying healthcare-associated infections.[35]

**Sample name**	**Year**	**Sample type**	**Ward**	**Hospital**	**Date**	accession	ENA biosample accession
NCTC11045	1975	Reference	Reference	Reference	01/01/1975	ERS20245094	SAMEA115735665
DSM67173	1991	Reference	Reference	Reference	01/01/1991	ERS20245095	SAMEA115735666
Sample_1	2017	Blood	NICU	Hospital 1	20/04/2017	ERS20245096	SAMEA115735667
Sample_2	2017	Blood	NICU	Hospital 1	15/05/2017	ERS20245097	SAMEA115735668
Sample_3	2018	Blood	Other_adult	Hospital 1	26/06/2018	ERS20245098	SAMEA115735669
Sample_4	2018	Blood	Other_adult	Hospital 1	14/12/2018	ERS20245099	SAMEA115735670
Sample_5	2019	CSF	NICU	Hospital 1	20/04/2019	ERS20245100	SAMEA115735671
Sample_6	2019	Blood	NICU	Hospital 1	16/07/2019	ERS20245101	SAMEA115735672
Sample_7	2020	Blood	NICU	Hospital 1	02/06/2020	ERS20245102	SAMEA115735673
Sample_8	2020	Blood	NICU	Hospital 1	04/06/2020	ERS20245103	SAMEA115735674
Sample_9	2020	Blood	NICU	Hospital 1	11/06/2020	ERS20245104	SAMEA115735675
Sample_10	2020	Blood	NICU	Hospital 1	17/06/2020	ERS20245105	SAMEA115735676
Sample_11	2020	Blood	NICU	Hospital 1	09/07/2020	ERS20245106	SAMEA115735677
Sample_12	2020	Blood	NICU	Hospital 1	11/07/2020	ERS20245107	SAMEA115735678
Sample_13	2020	Blood	NICU	Hospital 1	11/07/2020	ERS20245108	SAMEA115735679
Sample_14	2020	Line	NICU	Hospital 1	24/09/2020	ERS20245109	SAMEA115735680
Sample_15	2020	Blood	NICU	Hospital 1	19/11/2020	ERS20245110	SAMEA115735681
Sample_16	2020	Blood	AICU	Hospital 1	20/11/2020	ERS20245111	SAMEA115735682
Sample_17	2020	Blood	NICU	Hospital 1	08/12/2020	ERS20245112	SAMEA115735683
Sample_18	2021	Blood	NICU	Hospital 1	04/01/2021	ERS20245113	SAMEA115735684
Sample_19	2021	Blood	NICU	Hospital 1	24/03/2021	ERS20245114	SAMEA115735685
Sample_20	2021	Line	NICU	Hospital 1	25/03/2021	ERS20245115	SAMEA115735686
Sample_21	2021	Line	NICU	Hospital 1	31/03/2021	ERS20245116	SAMEA115735687
Sample_23	2021	Blood	NICU	Hospital 2	01/04/2021	ERS20245117	SAMEA115735688
Sample_22	2021	Blood	Other_paediatric	Hospital 2	01/04/2021	ERS20245118	SAMEA115735689
Sample_24	2021	Blood	NICU	Hospital 1	04/04/2021	ERS20245119	SAMEA115735690
Sample_25	2021	Blood	NICU	Hospital 1	13/04/2021	ERS20245120	SAMEA115735691
Sample_26	2021	Blood	Other_adult	Hospital 1	04/08/2021	ERS20245121	SAMEA115735692
Sample_27	2021	Blood	Other_adult	Hospital 1	06/08/2021	ERS20245122	SAMEA115735693
Sample_28	2022	SFC	NICU	Hospital 1	10/03/2022	ERS20245123	SAMEA115735694
Sample_29	2022	Blood	NICU	Hospital 1	07/04/2022	ERS20245124	SAMEA115735695
Environment_1	2022	Environment	NICU	Hospital 1	21/04/2022	ERS20245125	SAMEA115735696
Environment_2	2022	Environment	NICU	Hospital 1	21/04/2022	ERS20245126	SAMEA115735697
Environment_3	2022	Environment	NICU	Hospital 1	21/04/2022	ERS20245127	SAMEA115735698
Environment_4	2022	Environment	NICU	Hospital 1	21/04/2022	ERS20245128	SAMEA115735699
Environment_5	2022	Environment	NICU	Hospital 1	21/04/2022	ERS20245129	SAMEA115735700
Sample_30	2022	Blood	NICU	Hospital 1	30/04/2022	ERS20245130	SAMEA115735701
Sample_31	2022	SFC	NICU	Hospital 1	01/05/2022	ERS20245131	SAMEA115735702
Sample_32	2022	Blood	NICU	Hospital 1	06/05/2022	ERS20245132	SAMEA115735703
Sample_33	2022	Blood	NICU	Hospital 1	08/05/2022	ERS20245133	SAMEA115735704

Sample_34	2022	Blood	NICU	Hospital 1	16/05/2022	ERS20245134	SAMEA115735705
Sample_35	2022	Rectal	NICU	Hospital 1	16/05/2022	ERS20245135	SAMEA115735706
Sample_36	2022	SFC	NICU	Hospital 1	17/05/2022	ERS20245136	SAMEA115735707
Sample_37	2022	Rectal	NICU	Hospital 1	23/05/2022	ERS20245137	SAMEA115735708
Sample_38	2022	Blood	NICU	Hospital 1	02/06/2022	ERS20245138	SAMEA115735709
Sample_39	2022	SFC	NICU	Hospital 1	12/06/2022	ERS20245139	SAMEA115735710
Sample_40	2022	SFC	NICU	Hospital 1	13/06/2022	ERS20245140	SAMEA115735711

Table S5: list of samples included in sequence analysis with metadata including accession numbers for short read archive

SUPPLEMENTARY FIGURES:

Figure S1: Timeline of positive *S. capitis* and CoNS samples included in demographic & clinical analysis

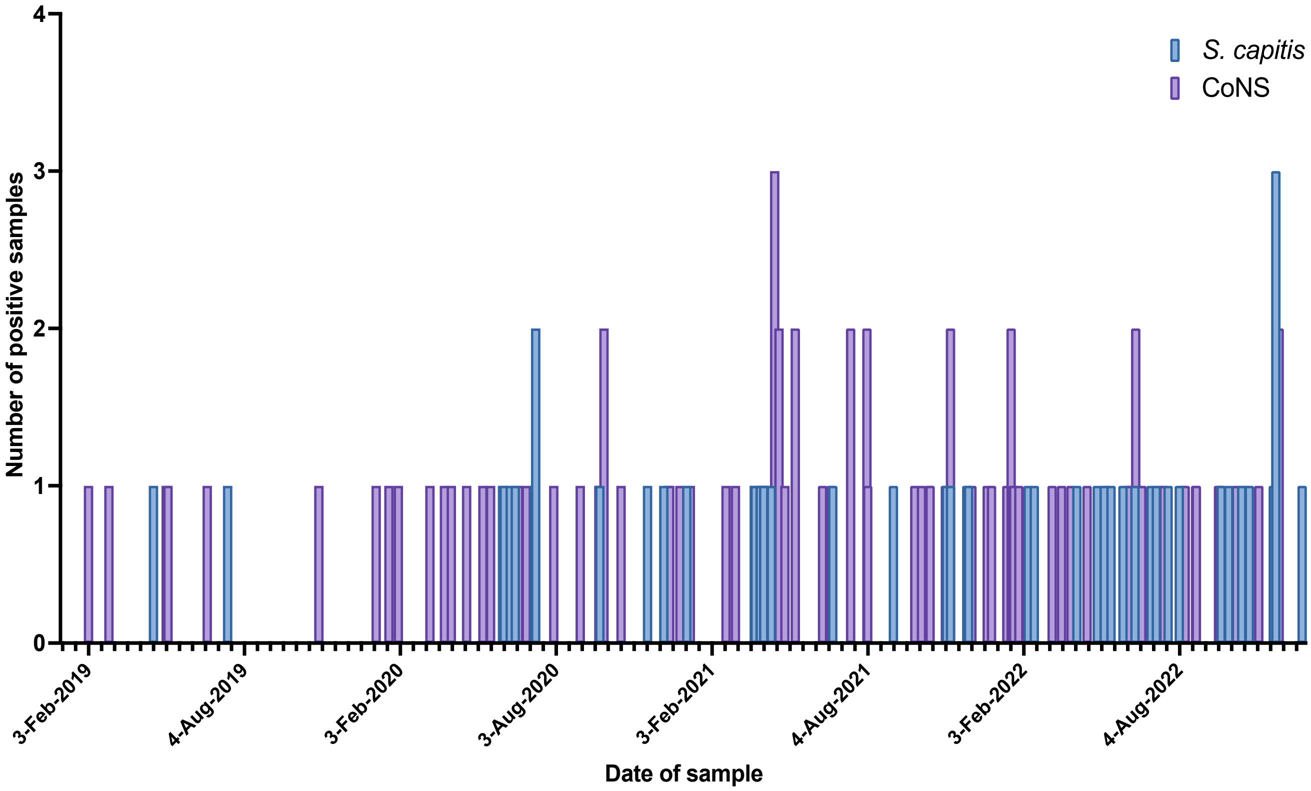

Figure S2: Incubator locations sampled[36,37]

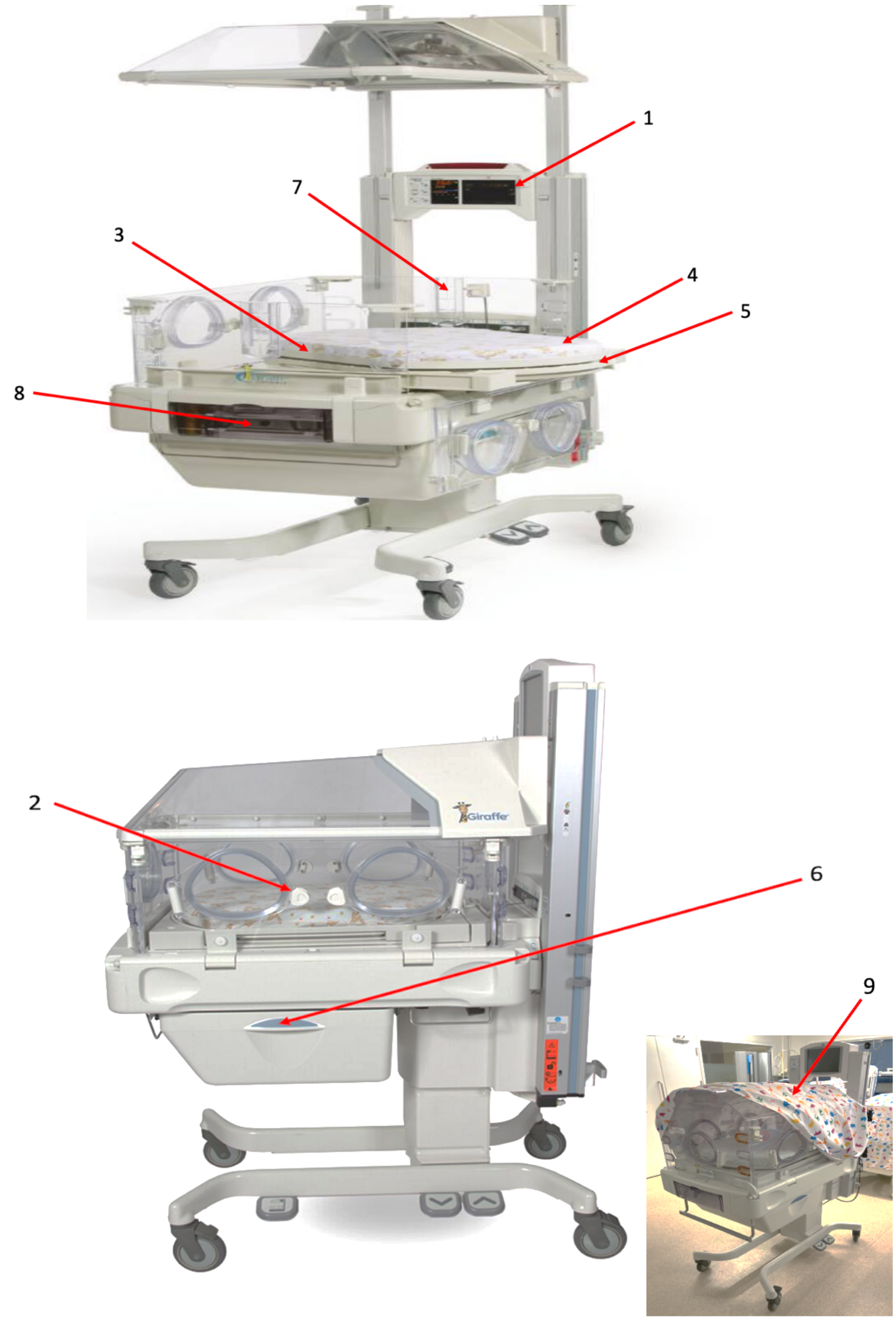

KEY:

No.	Location	Swab	Aerobic (AC) Petrifilm™	Staph Express (SE) Petrifilm™
**1**	Control panel	Y	Y	Y
**2**	Door handles x 4 (use 1 swab/Petrifilm for the 2 handles on each side)	Y (x2)	Y	Y
**3**	Beneath mattress	Y	Y	Y
**4**	Mattress	Y		
**5**	Gap around inbuilt weighing scale	Y		
**6**	Handle of drawer beneath incubator	Y	Y	Y
**7**	Area where ventilatory tubing enters incubator	Y		
**8**	Air filter behind water container	Y	Y	Y
**9**	Fabric cover overlaying incubator (inset)	Y		

NB: 2x clean and 2x in-use incubators sampled

Figure S3: NICU layout and additional locations sampled

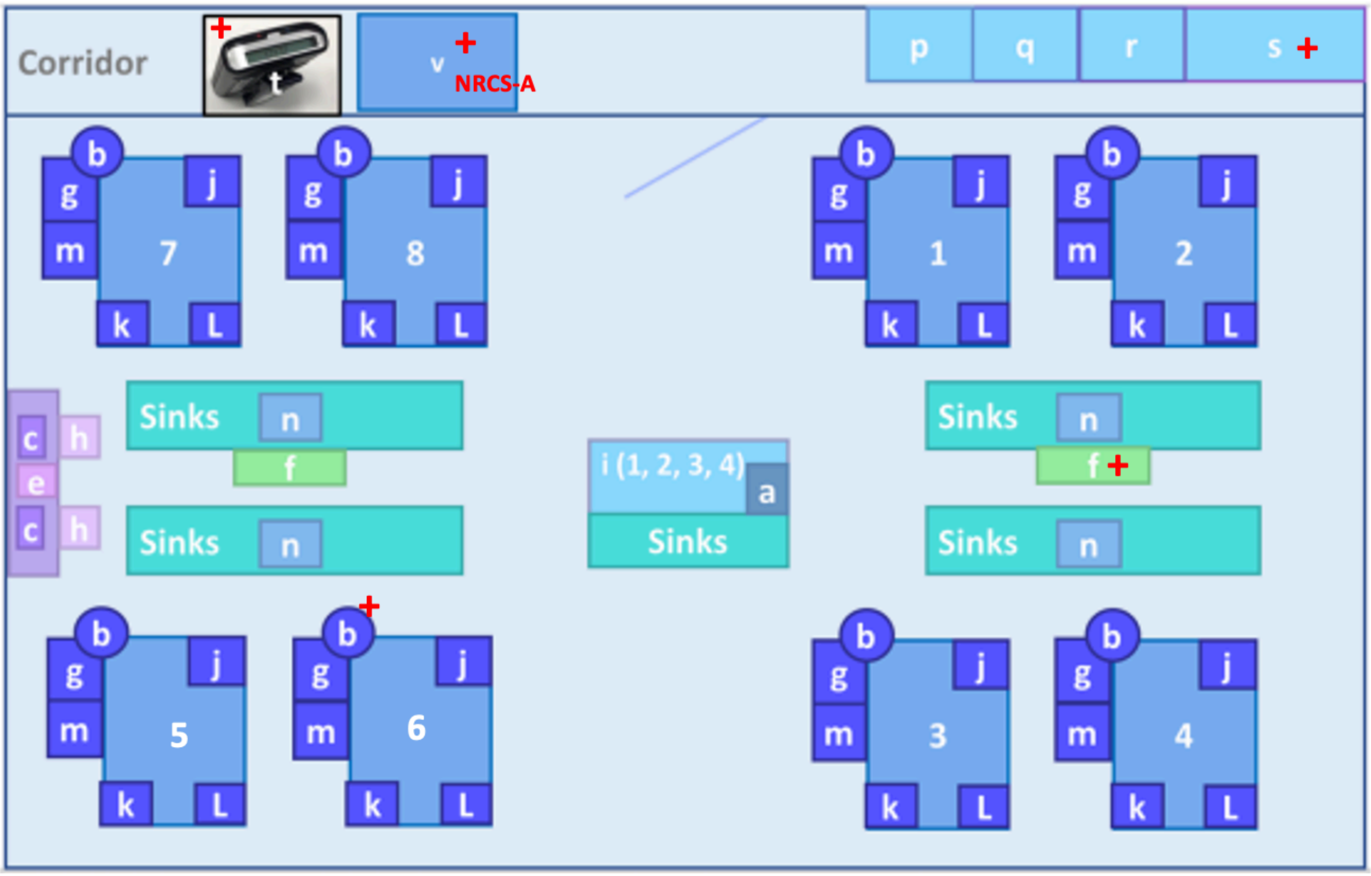

Notes:

- *In-use incubators sampled were 6 & 7*
- *+ **denotes isolation of S. capitis from this site:** Doctors' pager, door handle of clean incubator, handle of store cupboard, stethoscope of in-use incubator, notes trolley. NRCS-A clone was isolated from a clean incubator door handle*

KEY:

**Code**	**Location**	**Sample type**
**a**	Nappy scale	AC Petrifilm™ + swab
**b**	Stethoscope (attached to each incubator; used for one baby)	Swab
**c**	Computer keyboard	Swab
**d**	Mobile telephones (staff x2 – not pictured)	AC Petrifilm™ + swab
**e**	Static telephone	Swab
**f**	Notes trolley handle (x2)	AC Petrifilm™ + swab
**g**	Parental chair next to incubator	Swab
**h**	Staff chairs by computers	Swab
**i**	Drawers of equipment trolley (containing frequently used items – e.g. feeding tubes, nappies)	AC Petrifilm™ + swab
**j**	Nursing notes station (located next to each incubator)	AC Petrifilm™ + swab
**k**	Silencing alarm on vital signs monitor (next to each incubator)	AC Petrifilm™ + swab
**l**	Infusion pump alarm buttons (pumps next to each incubator)	AC Petrifilm™ + swab
**m**	Breast pump (next to each incubator)	Swab
**n**	Blue trays (used to gather equipment for venepuncture and taken to incubator x4)	AC Petrifilm™ + swab
**o**	Sluice (location incubators are cleaned: sink x1, worktop x1, drawer handle x1)	Swab
**p**	Cardiac echo machine (probe)	AC Petrifilm™ and swab
**q**	Cranial ultrasound machine (probe + keyboard)	AC Petrifilm™ and swab
**r**	X-ray plate from portable x-ray machine (placed in slot beneath incubators)	AC Petrifilm™ + swab
**s**	Handle of store cupboard in corridor	Swab
**t**	Doctors' pager (baton pager shared between multiple staff)	Swab
**v**	Clean incubator door handle	AC Petrifilm™, SE Petrifilm™ and swab

Figure S4: CoNS samples in control bacteraemia episodes by (**a**) species and (**b**) infection type.

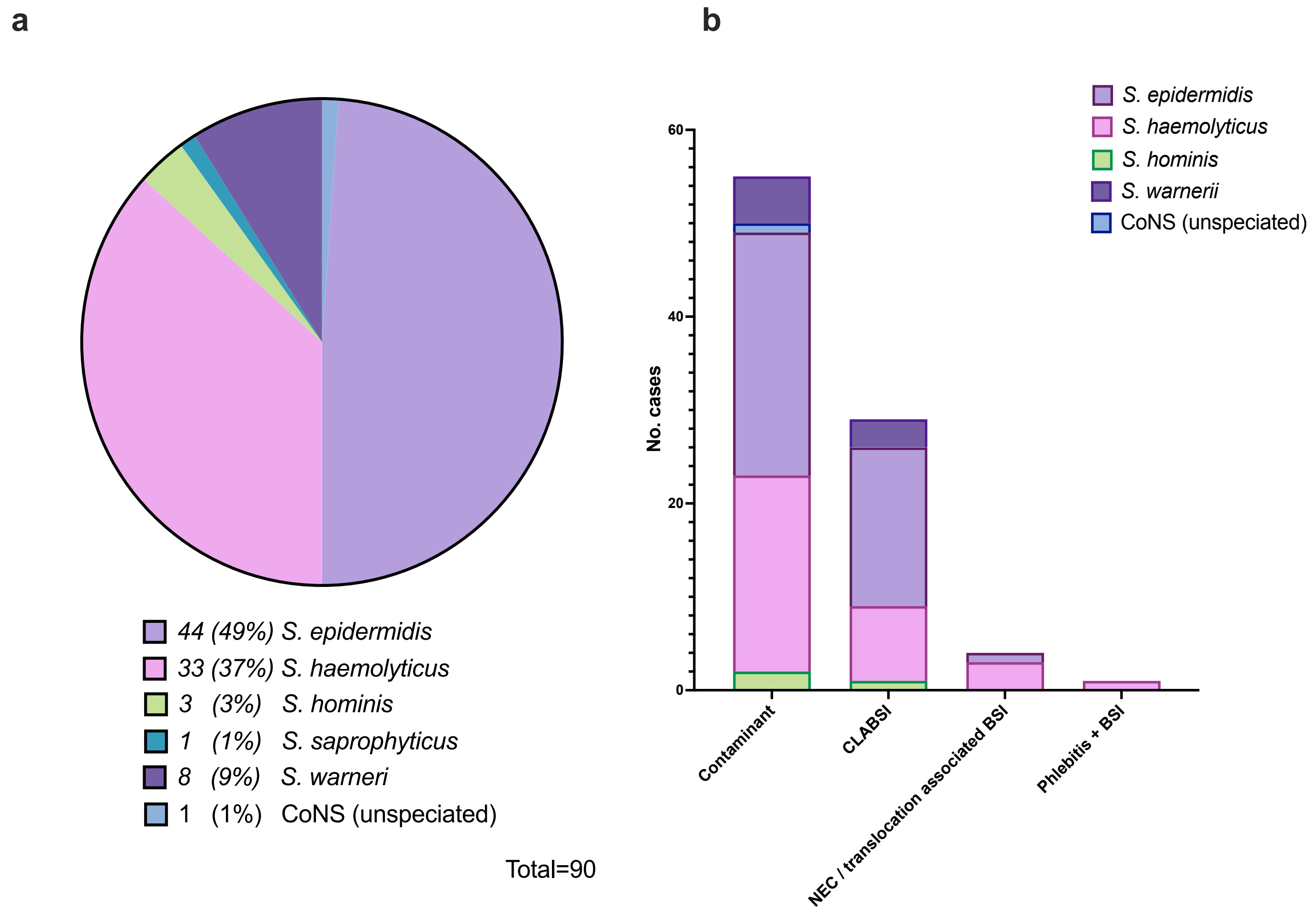

Key: Samples listed as contaminant were adjudicated not to be representative of a true infection.
NB: Sample listed as unspeciated CoNS had been speciated via MALDI-TOF MS Biotyper and was verified to be non-*Capitis* isolate, however, the identity of the organism was not recorded in Hospital 1's microbiological reporting system.

Figure S5: Staphylococcal species in sterile sample by (**a**) Gestation, (**b**) Birthweight and (**c**) Days of age at sample. Staphylococcal species in sterile sample adjudicated as infection by (**d**) Gestation, (**e**) Birthweight and (**f**) Days of age at sample

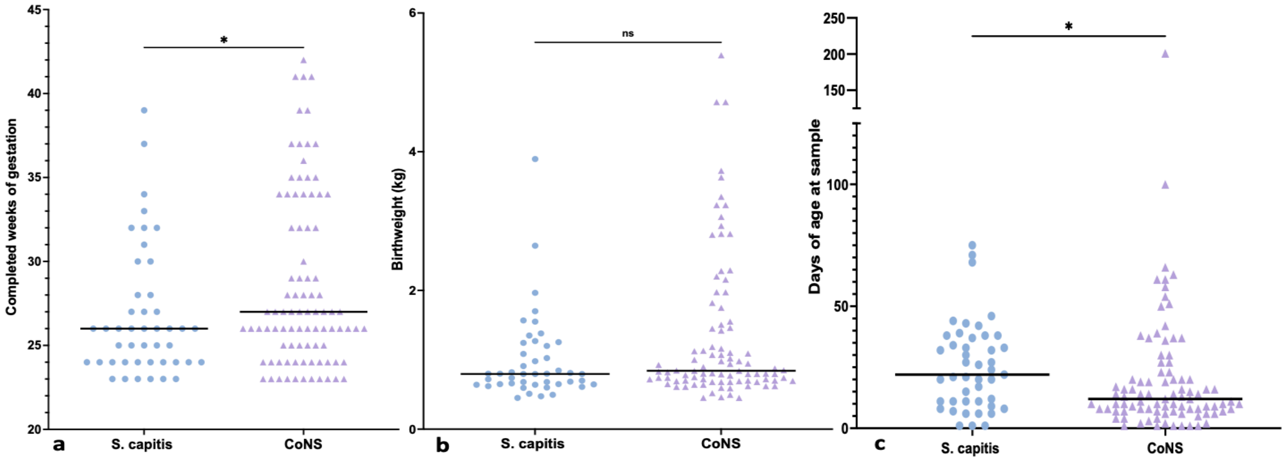

Key: * = p<0.05, ns = not significant

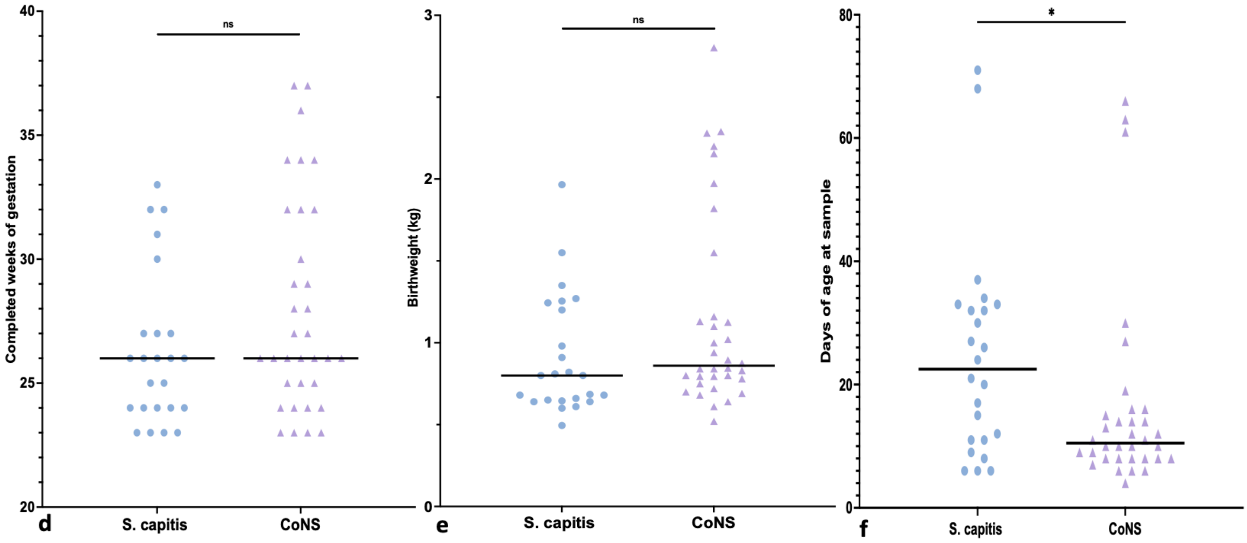

Key: * = p<0.05, ns = not significant

Figure S6: Phylogenetic tree of short read sequencing alignments of local NICU isolates and 2 reference strains, compared with published NRCS-A WGS assemblies originating from UK (CR05), France (CR01 and CR09), Belgium (CR03) and Australia (CR04).

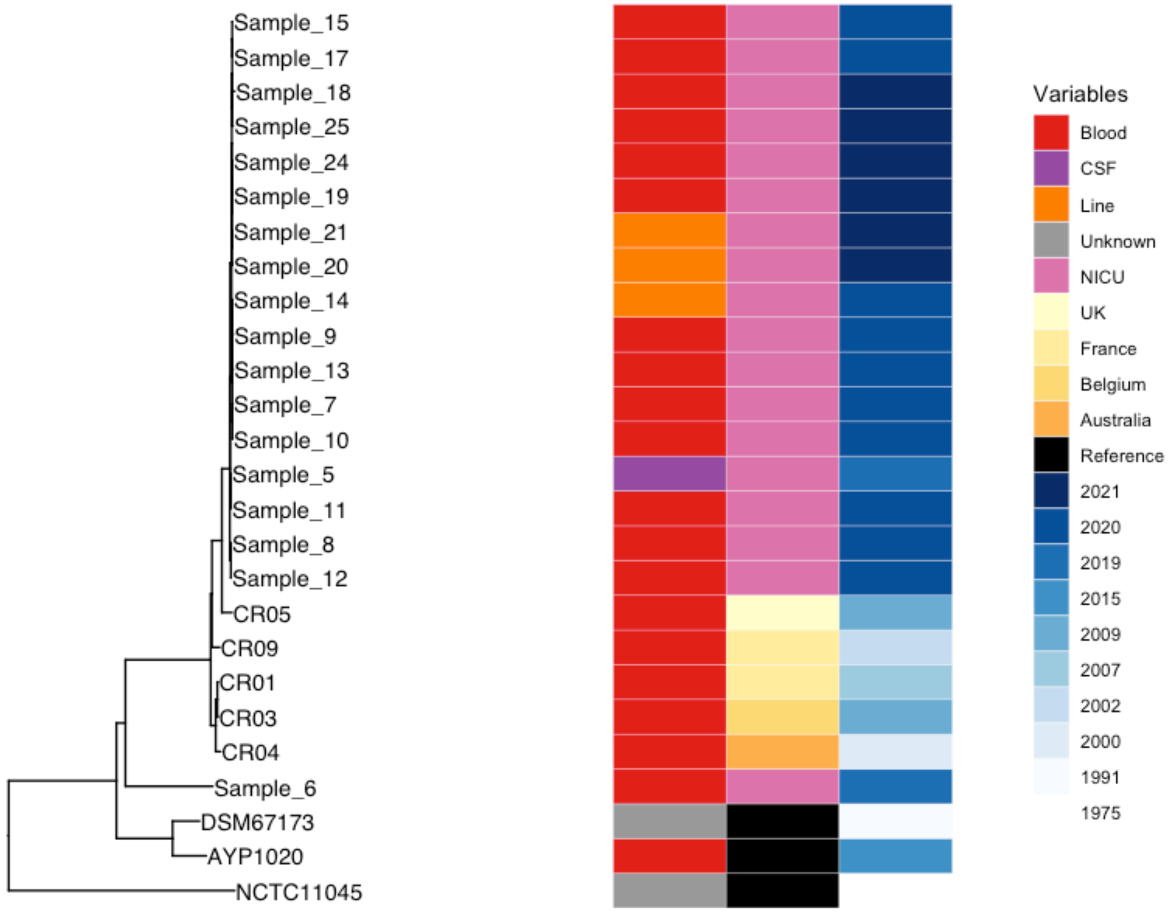

Figure S7: Time-dated phylogenetic tree of NRCS-A cluster *S. capitis* isolates in SRA (grey) and from the present study (blue), after adjustment for recombination. 95% confidence intervals for time of most recent common ancestor is marked by horizontal red bars.

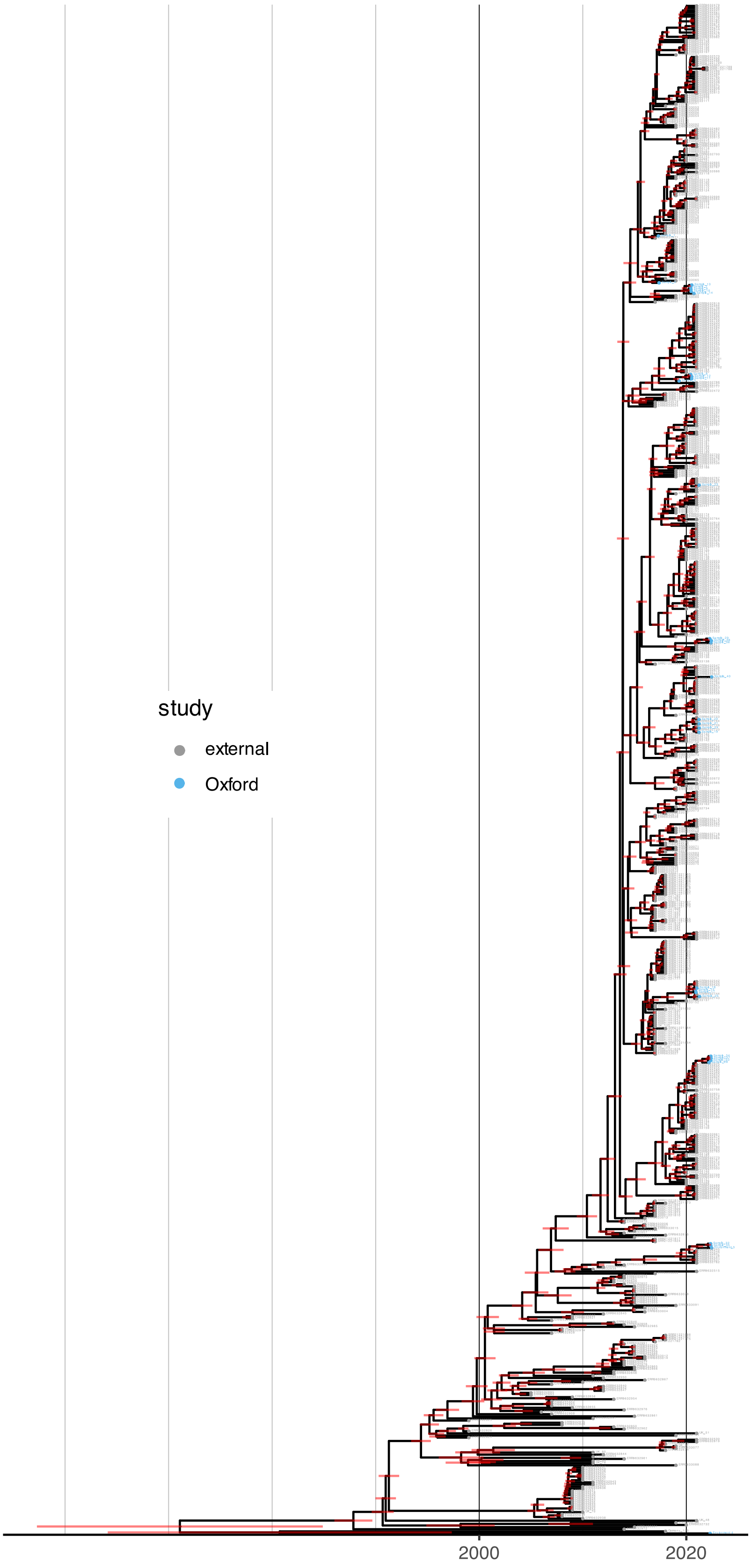

Figure S8 : Time -dated phylogenetic tree, adjusted for recombination, comparing NRCS-A samples from this study (blue) and externally sequenced *S. capitis* retrieved from the NCBI SRA (red). Tips have been sub-sampled to reduce oversampling. 95% confidence intervals for time of most recent common ancestor are marked by horizontal red bars

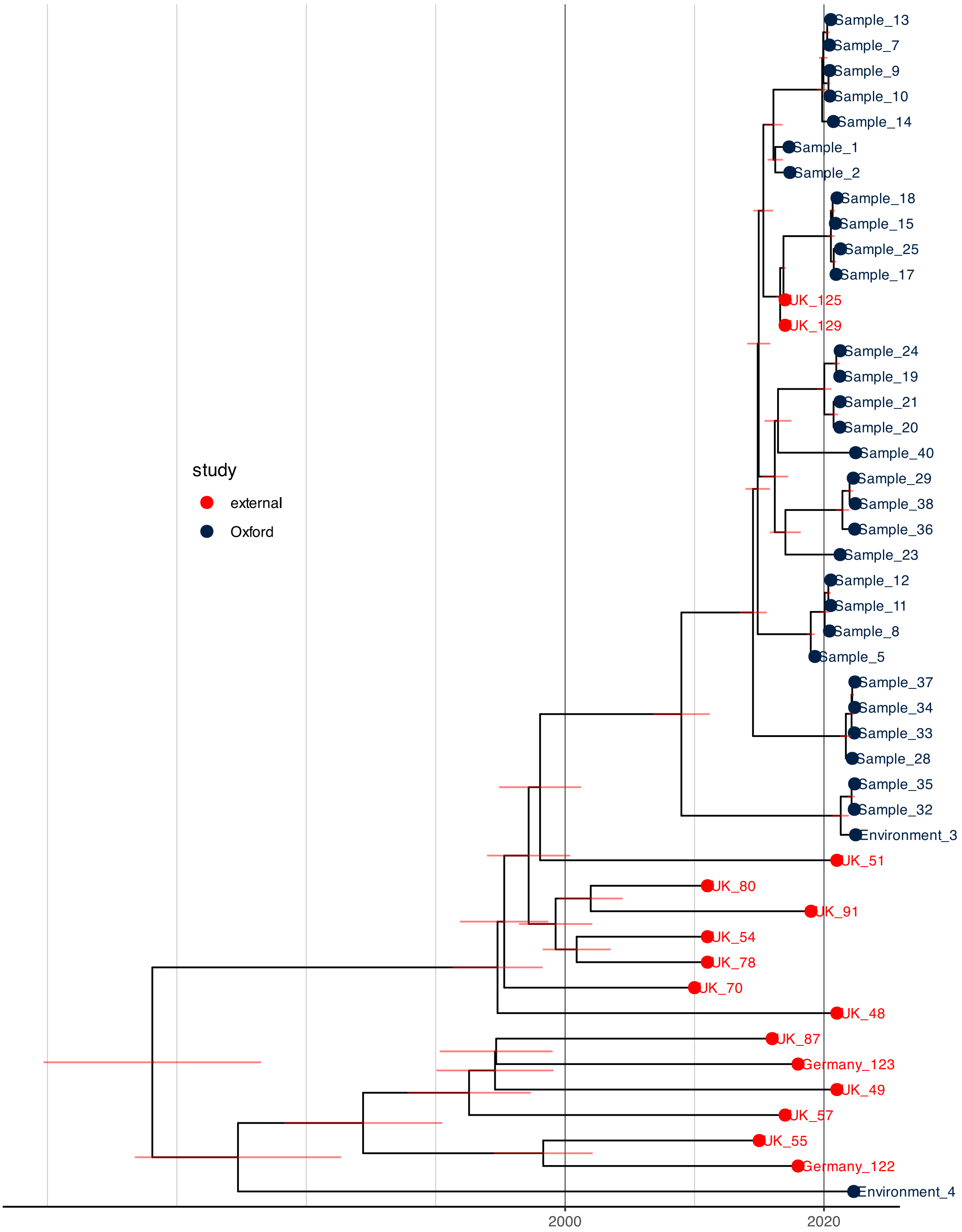

Figure S9 Phylogenetic distance over SNP distance for each pair of leaves in recombination adjusted phylogeny Figure S8.

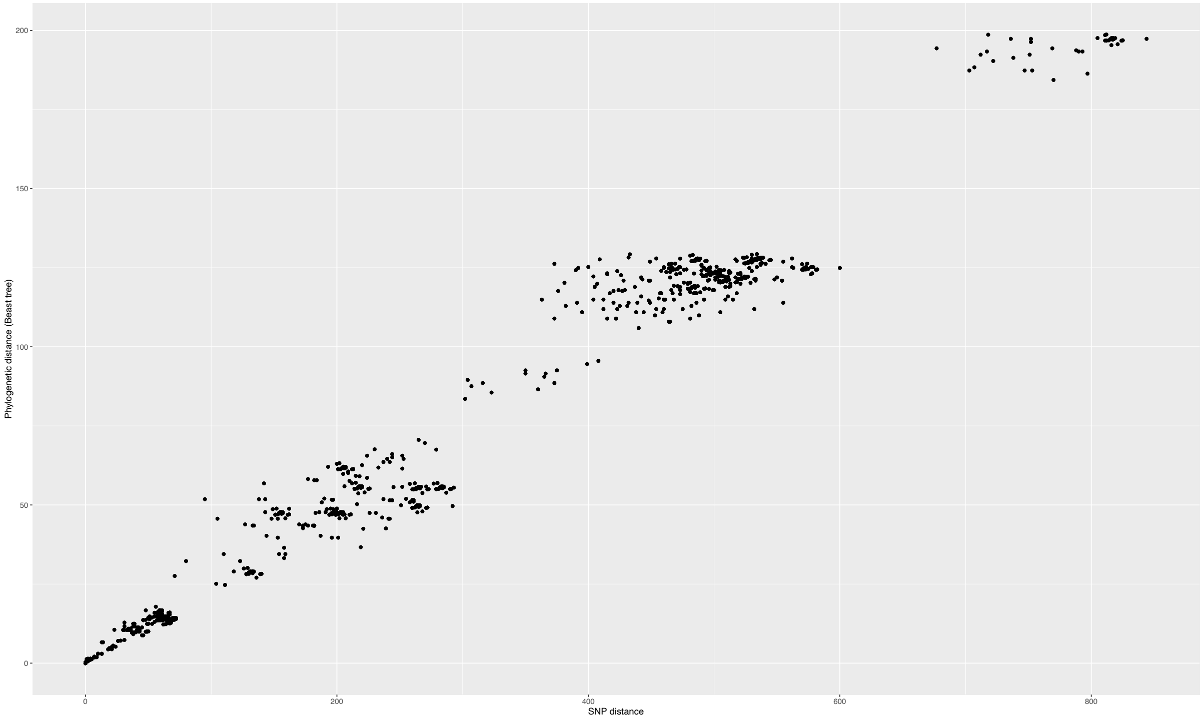

Figure S10 Pairwise distance matrix of 33 closely related *S. capitis* strains from clinical and environmental samples found in NICU (all containing *nsr* gene). Sample 39 and Environment 4 (from a notes trolley) show genomic distances over 500 SNPs from the most closely related isolates in the NICU cluster.

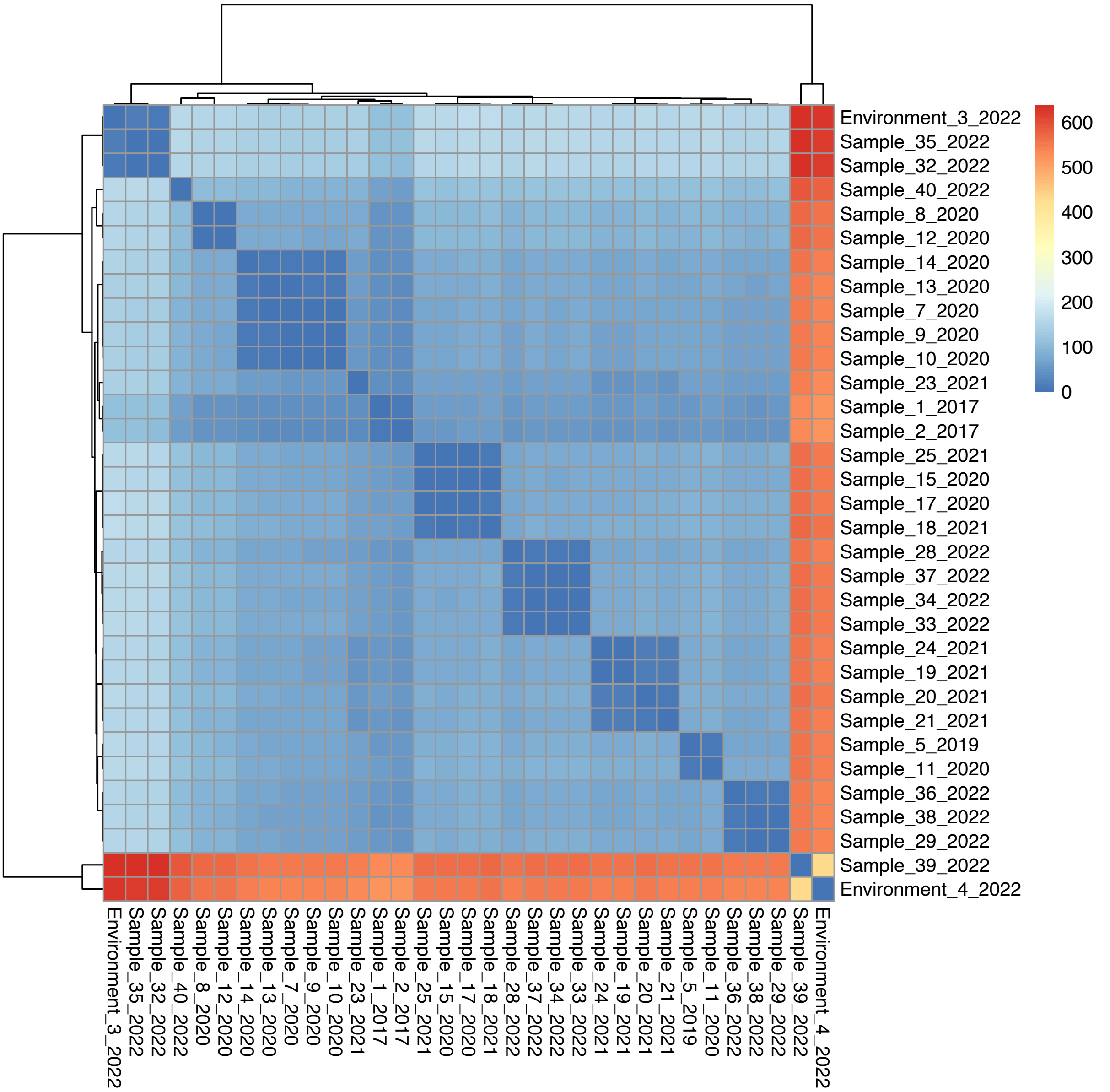

Figure S11 Timeline of admission, rectal and blood culture results for two infants with *S. capitis* detected in rectal swabs and sterile site samples

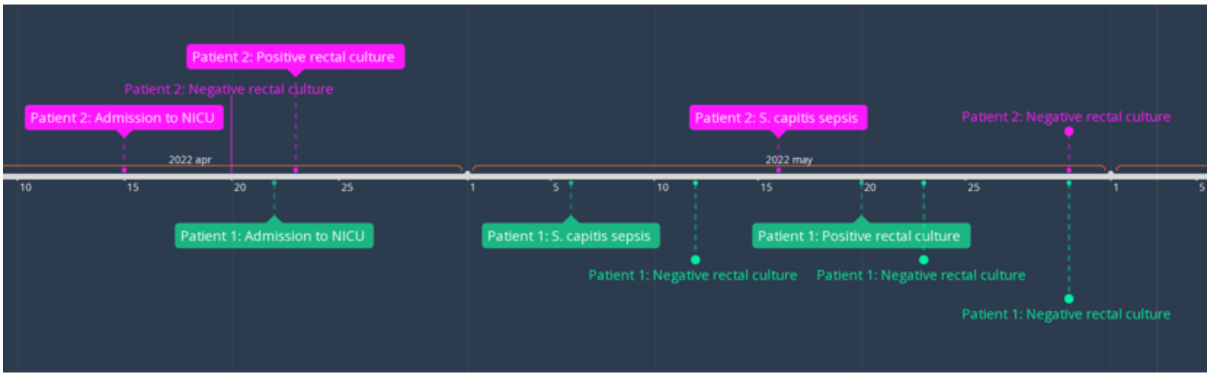

References:

1. QIAGEN. QIAGEN® Genomic DNA Preparation, 2012. QIAGEN-Genomic-DNA-Preparation--April-2012-EN.pdf. Published 2012. Accessed 21st February 2022.
2. Illumina. MiSeq System Denature and Dilute Libraries Guide. https://support.illumina.com/content/dam/illumina-support/documents/documentation/system_documentation/miseq/miseq-denature-dilute-libraries-guide-15039740-10.pdf. Published 2019. Accessed 21st February 2021.
3. Illumina. Illumina DNA Prep Reference Guide. https://emea.support.illumina.com/content/dam/illumina-support/documents/documentation/chemistry_documentation/illumina_prep/illumina-dna-prep-reference-guide-1000000025416-09.pdf. Published 2020. Accessed 21st February 2021.
4. Guppy basecaller, Version 5. https://nanoporetech.com/community.
5. ModernisingMedicalMicrobiology. Compass Compact. https://github.com/oxfordmmm/CompassCompact. Published 2019. Accessed 23rd February, 2023.
6. Zerbino DR, Birney E. Velvet: algorithms for de novo short read assembly using de Bruijn graphs. *Genome Res.* 2008;18(5):821-829.
7. Lemriss H, Lemriss S, Martins-Simoes P, et al. Genome Sequences of Four Staphylococcus capitis NRCS-A Isolates from Geographically Distant Neonatal Intensive Care Units. *Genome Announc.* 2015;3(4).
8. Lemriss H, Martins Simoes P, Lemriss S, et al. Non-contiguous finished genome sequence of Staphylococcus capitis CR01 (pulsetype NRCS-A). *Stand Genomic Sci.* 2014;9(3):1118-1127.
9. Criscuolo A. On the transformation of MinHash-based uncorrected distances into proper evolutionary distances for phylogenetic inference [version 1; peer review: 3 approved]. *F1000Research 2020.* 2020;9:1309
10. Petit R. dragonflye. https://github.com/rpetit3/dragonflye#software-included-19. Published 2023. Accessed 23rd October, 2023.
11. Kolmogorov M, Yuan J, Lin Y, Pevzner PA. Assembly of long, error-prone reads using repeat graphs. *Nat Biotechnol.* 2019;37(5):540-546.
12. Vaser R, Sovic I, Nagarajan N, Sikic M. Fast and accurate de novo genome assembly from long uncorrected reads. *Genome Res.* 2017;27(5):737-746.
13. Wick RR, Holt KE. Polypolish: Short-read polishing of long-read bacterial genome assemblies. *PLoS Comput Biol.* 2022;18(1):e1009802.
14. Walker BJ, Abeel T, Shea T, et al. Pilon: an integrated tool for comprehensive microbial variant detection and genome assembly improvement. *PLoS One.* 2014;9(11):e112963.
15. Sanderson ND, Street TL, Foster D, et al. Real-time analysis of nanopore-based metagenomic sequencing from infected orthopaedic devices. *BMC Genomics.* 2018;19(1):714.

16. Kim D, Song L, Breitwieser FP, Salzberg SL. Centrifuge: rapid and sensitive classification of metagenomic sequences. *Genome Res.* 2016;26(12):1721-1729.
17. Li H. Minimap2: pairwise alignment for nucleotide sequences. *Bioinformatics.* 2018;34(18):3094-3100.
18. *Vulnerability Static Analysis for Containers* [computer program]. 2019. https://github.com/quay/clair. Accessed 31st May 2024.
19. Sanderson ND, Swann J, Barker L, et al. High precision Neisseria gonorrhoeae variant and antimicrobial resistance calling from metagenomic Nanopore sequencing. *Genome Res.* 2020;30(9):1354-1363.
20. ModernisingMedicalMicrobiology. genericbugontworkflow. https://gitlab.com/ModernisingMedicalMicrobiology/genericbugontworkflow. Accessed 23rd February 2023.
21. Stamatakis A. RAxML version 8: a tool for phylogenetic analysis and post-analysis of large phylogenies. *Bioinformatics.* 2014;30(9):1312-1313.
22. Didelot X, Wilson DJ. ClonalFrameML: efficient inference of recombination in whole bacterial genomes. *PLoS Comput Biol.* 2015;11(2):e1004041.
23. Wan Y, Ganner M, Mumin Z, et al. Whole-genome sequencing reveals widespread presence of Staphylococcus capitis NRCS-A clone in neonatal units across the United Kingdom. *J Infect.* 2023;87(3):210-219.
24. Felgate H, Sethi D, Faust K, et al. Characterisation of neonatal Staphylococcus capitis NRCS-A isolates compared with non NRCS-A Staphylococcus capitis from neonates and adults. *Microb Genom.* 2023;9(10).
25. Seemann T. SNIPPY: fast bacterial variant calling from NGS reads 2015. https://github.com/tseemann/snippy. Accessed 31st May 2024.
26. ModernisingMedicalMicrobiology. https://github.com/oxfordmmm/Illumina_SNIPPY_workflow.
27. Menardo F, Loiseau C, Brites D, et al. Treemmer: a tool to reduce large phylogenetic datasets with minimal loss of diversity. *BMC Bioinformatics.* 2018;19(1):164.
28. Eyre D, Constantinides B. runListCompare. https://github.com/davideyre/runListCompare. Accessed 31st May 2024.
29. Minh BQ, Schmidt HA, Chernomor O, et al. IQ-TREE 2: New Models and Efficient Methods for Phylogenetic Inference in the Genomic Era. *Mol Biol Evol*. 2020 May 1;37(5):1530-1534. doi: 10.1093/molbev/msaa015.
30. Suchard MA, Lemey P, Baele G, Ayres DL, Drummond AJ, Rambaut A. Bayesian phylogenetic and phylodynamic data integration using BEAST 1.10. *Virus Evol.* 2018;4(1):vey016.
31. Bouckaert R, Vaughan TG, Barido-Sottani J, et al. BEAST 2.5: An advanced software platform for Bayesian evolutionary analysis. *PLoS Comput Biol.* 2019;15(4):e1006650.
32. https://github.com/oxfordmmm/pathogen_phylogenetics_pipeline
33. *Prism Version 9.5.0* [computer program]. San Diego, California USA2023.
34. Public Health England Infection in Critical Care Quality Improvement Programme. Surveillance of Blood Stream Infections in Patients Attending ICUs in England: Protocol version 3.4. https://www.ficm.ac.uk/sites/ficm/files/documents/2021-10/protocol_v3.4_07082018.pdf. Published 2017. Accessed 17th October, 2023.
35. National Healthcare Safety Network (NHSN). Bloodstream Infection Event (Central Line-Associated Bloodstream Infection and Non-central Line Associated Bloodstream

Infection). https://www.cdc.gov/nhsn/pdfs/pscmanual/4psc_clabscurrent.pdf. Published 2023. Accessed 17th October, 2023.

36. wotol.com. Giraffe Incubator Carestation. https://www.wotol.com/product/ge-giraffe-omnibed-infant-warmer-and-incubator/2053415. Accessed 12th June 2024.

37. GE Healthcare. Giraffe Incubator Carestation. https://www.gehealthcare.co.uk/products/maternal-infant-care/giraffe-incubator-carestation. Accessed 12th June 2024.
